# Dataset of de novo assembly and functional annotation of the transcriptome during germination and initial growth of seedlings of *Myrciaria Dubia* “camu-camu”

**DOI:** 10.1016/j.dib.2020.105834

**Published:** 2020-06-11

**Authors:** Juan C. Castro, J. Dylan Maddox, Hicler N. Rodríguez, Carlos G. Castro, Sixto A. Imán-Correa, Marianela Cobos, Jae D. Paredes, Jorge L. Marapara, Janeth Braga, Pedro M. Adrianzén

**Affiliations:** a Unidad Especializada de Biotecnología, Centro de Investigaciones de Recursos Naturales de la Amazonía (CIRNA), Universidad Nacional de la Amazonia Peruana (UNAP), Psje. Los Paujiles S/N, San Juan Bautista, Loreto, 16000, Perú; b Departamento Académico de Ciencias Biomédicas y Biotecnología, Facultad de Ciencias Biológicas, Universidad Nacional de la Amazonia Peruana (UNAP), Ciudad Universitaria - Zungarococha, San Juan Bautista, Loreto, 16000, Perú; c Laboratorio de Biotecnología y Bioenergética (LBB), Universidad Científica del Perú (UCP), Av. Abelardo Quiñones km. 2.5, San Juan Bautista, Loreto, 16000 Perú; d Pritzker Laboratory for Molecular Systematics and Evolution, Field Museum of Natural History, 1400 S. Lake Shore Drive, Chicago, IL 60605, United States; e Environmental Sciences, American Public University System, Charles Town, WV 25414, United States; f Estación Experimental Agraria San Roque, Dirección de Recursos Genéticos y Biotecnología, Instituto Nacional de Innovación Agraria (INIA), Calle San Roque 236, San Juan Bautista, Loreto, 16000, Perú

**Keywords:** Gene expression, Germination, Metabolic pathways, Molecular sequence annotation, Plant development, RNA-seq, Seedlings

## Abstract

*Myrciaria dubia* “camu-camu” is a native shrub of the Amazon that is commonly found in areas that are flooded for three to four months during the annual hydrological cycle. This plant species is exceptional for its capacity to biosynthesize and accumulate important quantities of a variety of health-promoting phytochemicals, especially vitamin C [1], yet few genomic resources are available [2]. Here we provide the dataset of a de novo assembly and functional annotation of the transcriptome from a pool of samples obtained from seeds during the germination process and seedlings during the initial growth (until one month after germination). Total RNA/mRNA was purified from different types of plant materials (i.e., imbibited seeds, germinated seeds, and seedlings of one, two, three, and four weeks old), pooled in equimolar ratio to generate the cDNA library and RNA paired-end sequencing was conducted on an Illumina HiSeq™2500 platform. The transcriptome was de novo assembled using Trinity v2.9.1 and SuperTranscripts v2.9.1. A total of 21,161 transcripts were assembled ranging in size from 500 to 10,001 bp with a N50 value of 1,485 bp. Completeness of the assembly dataset was assessed using the Benchmarking Universal Single-Copy Orthologs (BUSCO) software v2/v3. Finally, the assembled transcripts were functionally annotated using TransDecoder v3.0.1 and the web-based platforms Kyoto Encyclopedia of Genes and Genomes (KEGG) Automatic Annotation Server (KAAS), and FunctionAnnotator. The raw reads were deposited into NCBI and are accessible via BioProject accession number PRJNA615000 (https://www.ncbi.nlm.nih.gov/bioproject/PRJNA615000) and Sequence Read Archive (SRA) with accession number SRX7990430 (https://www.ncbi.nlm.nih.gov/sra/SRX7990430). Additionally, transcriptome shotgun assembly sequences and functional annotations are available via Discover Mendeley Data (https://data.mendeley.com/datasets/2csj3h29fr/1).

Specifications table

**Table:** 

Subject	Genetics, Genomics and Molecular Biology
Specific subject area	Transcriptomics
Type of data	Figures, raw paired-end sequencing data, transcriptome shotgun assembly sequence database, and functional annotation results.
How data were acquired	Total RNA was isolated from seeds during the germination process and from seedlings during the initial growth (until one month after germination). High quality RNA samples were pooled and mRNA was purified. The library was constructed using standardized protocols and paired-end sequenced on an Illumina HiSeq™2500 platform.
Data format	Raw data in fastq format was deposited into NCBI database and available at BioProject accession number PRJNA615000 (https://www.ncbi.nlm.nih.gov/bioproject/PRJNA615000) and SRA accession number SRX7990430 (https://www.ncbi.nlm.nih.gov/sra/SRX7990430). Also, transcriptome shotgun assembly sequences database (fasta.gz format) and functional annotation results were deposited at Discover Mendeley Data (https://data.mendeley.com/datasets/2csj3h29fr/1).
Parameters for data collection	Total RNA was isolated from seeds during the germination process and from seedlings during the initial growth (until one month after germination). High quality RNA samples were pooled and mRNA was purified. The library was constructed using standardized protocols and paired-end sequenced on an Illumina HiSeq™2500 platform.
Description of data collection	Cleaned, high quality reads were de novo assembled with Trinity v2.9.1 and multiple gene transcripts combined into a single sequence with SuperTranscripts v2.9.1. Completeness of the assembly dataset was evaluated using the Benchmarking Universal Single-Copy Orthologs (BUSCO) software v2/v3 as implemented in the web-based server gVolante (https://gvolante.riken.jp/). The assembled transcripts were functionally annotated with TransDecoder v3.0.1, Kyoto Encyclopedia of Genes and Genomes (KEGG) Automatic Annotation Server (KAAS) v2.1 (https://www.genome.jp/tools/kaas/) and FunctionAnnotator (http://fa.cgu.edu.tw/index.php).
Data source location	Institution: Universidad Nacional de la Amazonia Peruana City/Town/Region: Iquitos/Maynas/Loreto Region Country: Peru Latitude and longitude (and GPS coordinates) for collected samples/data: *M. dubia* germplasm collection of the Instituto Nacional de Innovación Agraria (03°57′17′' S, 73°24′55′' W)
Data accessibility	Raw data in fastq format is available from NCBI under BioProject accession number PRJNA615000 (https://www.ncbi.nlm.nih.gov/bioproject/PRJNA615000) and SRA accession number SRX7990430 (https://www.ncbi.nlm.nih.gov/sra/SRX7990430). Transcriptome shotgun assembly sequence database (fasta.gz format) and functional annotations are hosted in the public repository Discover Mendeley Data (https://data.mendeley.com/datasets/2csj3h29fr/1).

## Value of the data

• This is the first dataset of the de novo assembly and functional transcriptome annotation during germination and initial growth of *M. dubia* seedlings.
• These data provide valuable information to elucidate the molecular mechanisms and genes involved in the complex process of germination, cell differentiation, and initial growth of seedlings of *M. dubia*.
• These data will allow further analysis to identify key genes involved in cellular differentiation and could provide the basis for the development of in vitro propagation protocols such as somatic embryogenesis of *M. dubia*.
• This transcriptome dataset can be used to elucidate the metabolic pathways involved in the biosynthesis of the variety of health-promoting phytochemicals produced and accumulated by *M. dubia*.

## 1 Data description

**Fig. 1:**
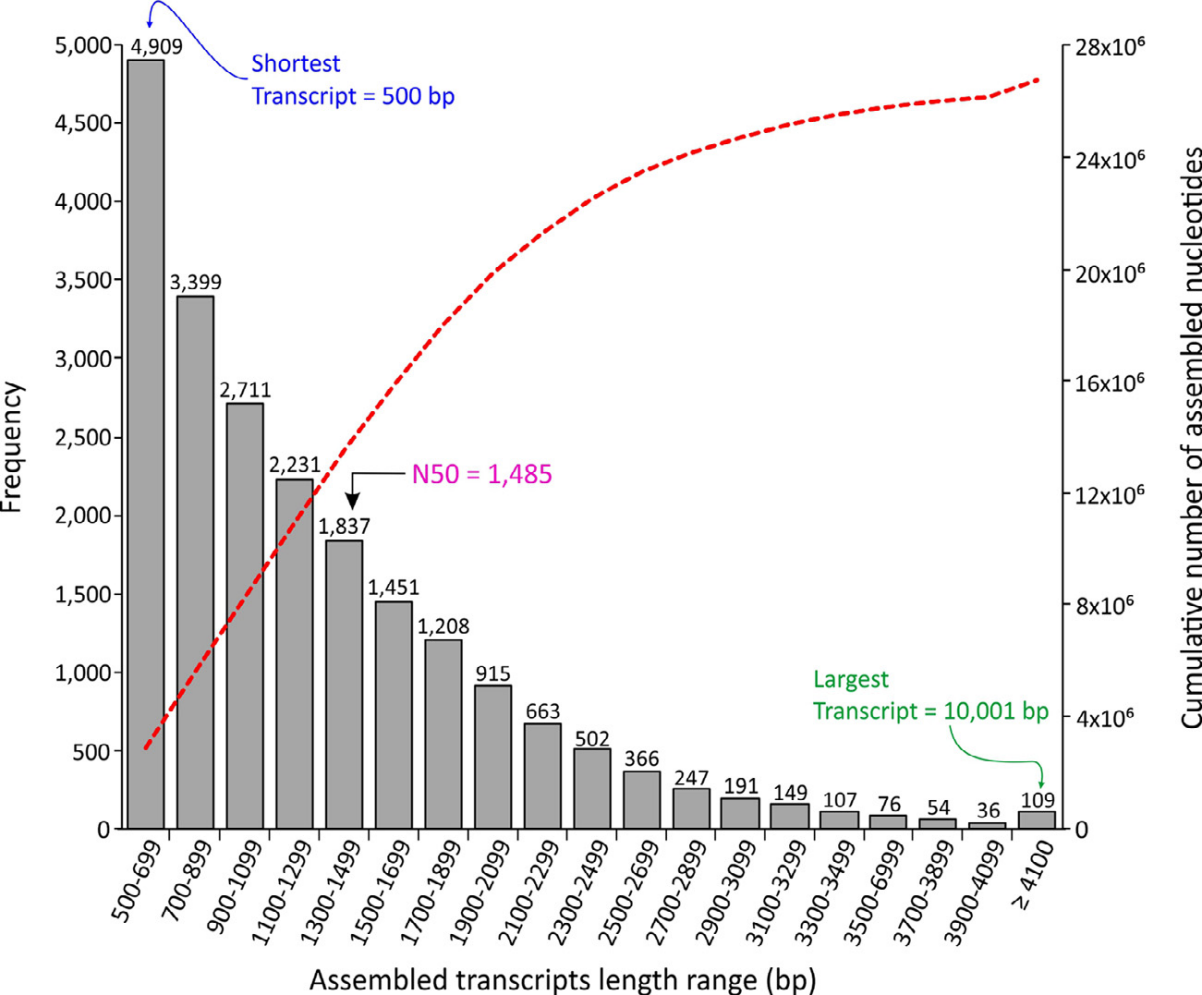
Distribution of the transcript lengths of the de novo assembled transcripts of the transcriptome obtained during germination and initial growth of seedlings of *M. dubia*.

**Fig. 2:**
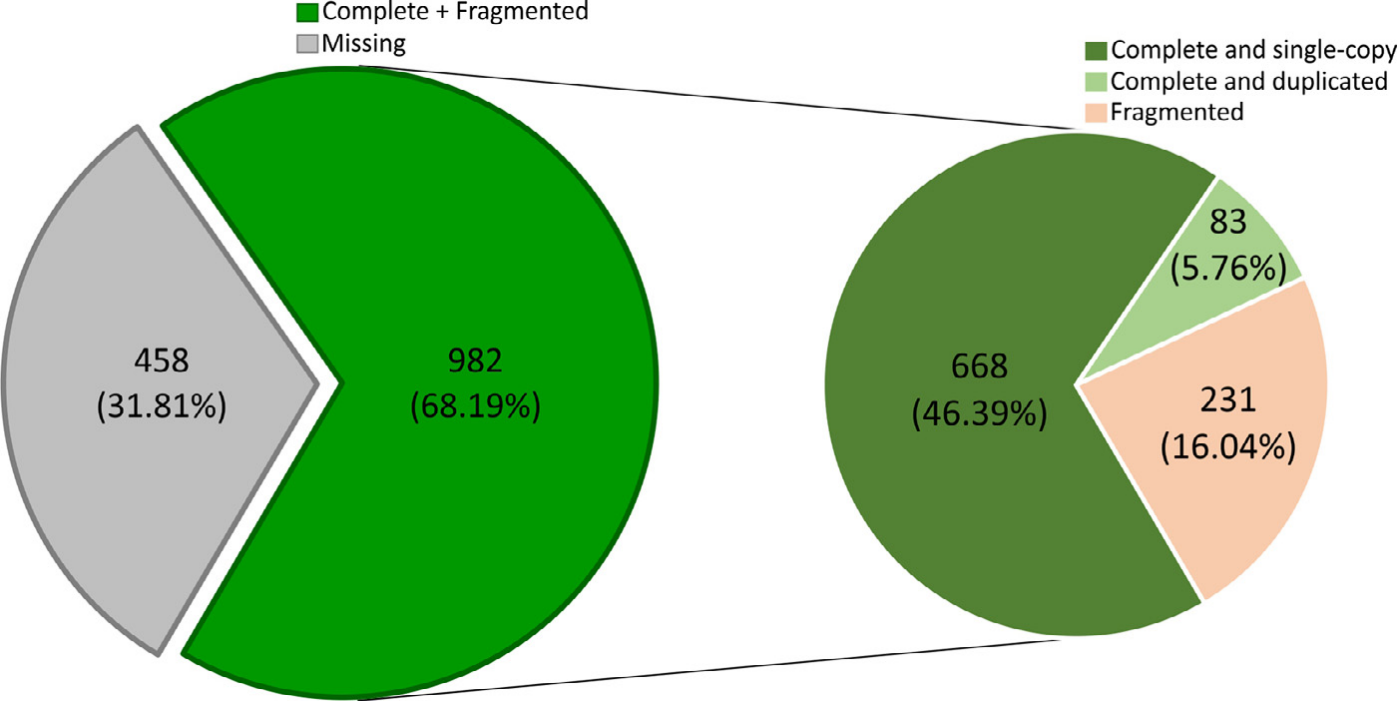
Completeness scores of the de novo assembled transcripts of the transcriptome obtained during germination and initial growth of seedlings of *M. dubia*.

**Fig. 3:**
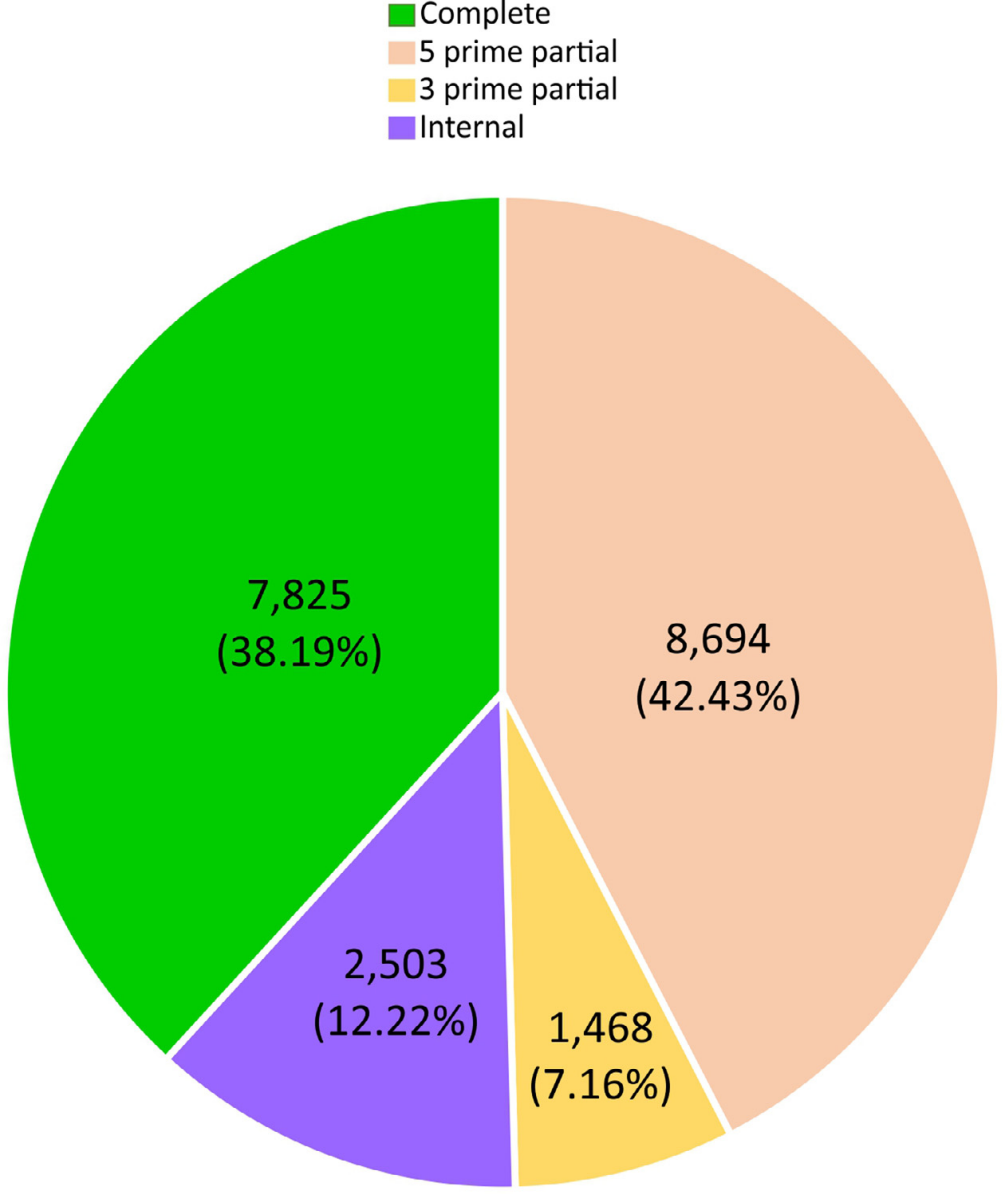
Summary of ORFs predicted in the de novo assembled transcripts of the transcriptome obtained during germination and initial growth of seedlings of *M. dubia*.

In this dataset the de novo assembly and functional annotation of the transcriptome during germination and initial growth of seedlings of *M. dubia* “camu-camu” is reported for the first time. Total RNA/mRNA from different types of plant materials (i.e., imbibited seeds, germinated seeds, and seedlings of one, two, three, and four weeks old) were pooled in equimolar ratios to construct the cDNA library and paired-end sequenced on an Illumina HiSeq™2500 platform. De novo transcriptome assembly was conducted using Trinity v2.9.1 and SuperTranscripts v2.9.1. In total, 21,161 transcripts with a range in size from 500 to 10,001 bp and N50 value of 1485 bp (Fig. 1) were assembled. Further, the completeness scores of the de novo assembled transcripts were evaluated using the Benchmarking Universal Single-Copy Orthologs (BUSCO) software, which revealed that of the 1440 core genes queried, 982 were detected (complete + partial = 68.19%) and 31.81% were missing (Fig. 2), with 46.39% of detected core genes that were complete and single copy (average number of orthologs per core genes = 1.13) Fig. 3.

**Fig. 4:**
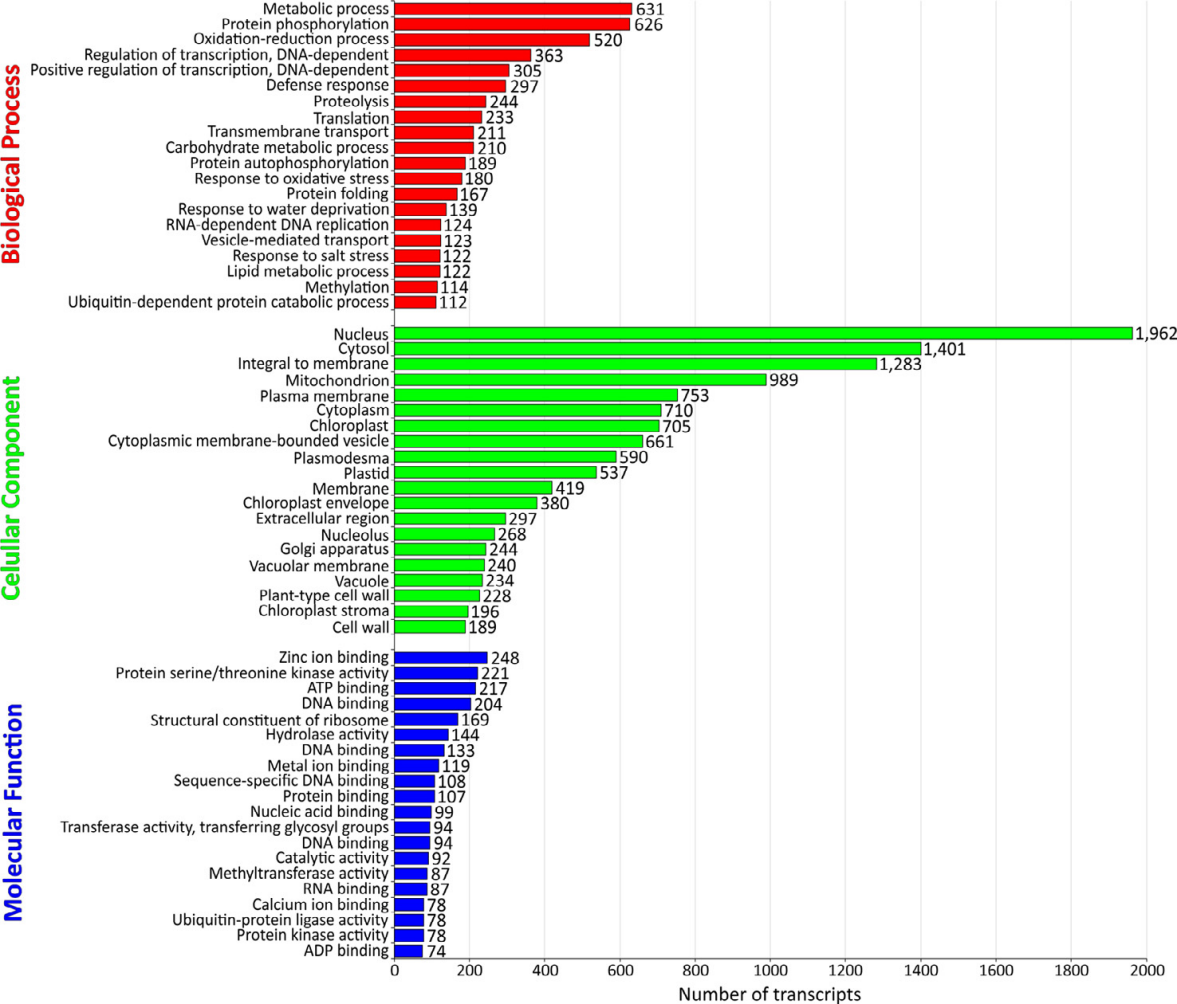
Gene Ontology classifications of the de novo assembled transcripts of the transcriptome obtained during germination and initial growth of seedlings of *M. dubia.*

The de novo assembled transcripts were also functionally annotated. First, Transdecoder predicted the four open reading frames categories as 38.19% complete, 42.43% 5 prime partial, 7.16% 3 prime partial and 12.22% internal. Second, Kyoto Encyclopedia of Genes and Genomes (KEGG) Automatic Annotation Server (KAAS) assigned KEGG Orthology (KO) IDs to 9043 transcripts with 3591 identified as unique. BRITE hierarchies (KEGG modules, KEGG orthology, and KEGG reaction modules) were also generated of which 162 metabolic pathway maps (with 3531 enzymes/proteins mapped) were related to plant metabolism and intracellular signaling such as ascorbate and aldarate metabolism (KEGG Pathway ID: 00053), photosynthesis (KEGG Pathway ID: 00195), carbon fixation in photosynthetic organisms (KEGG Pathway ID: 00710), phenylpropanoid biosynthesis (KEGG Pathway ID: 00940), plant-pathogen interaction (KEGG Pathway ID: 04626), among others (Table S1). Finally, FunctionAnnotator obtained 20,382 best hits from the NCBI non-redundant protein database with taxonomic distribution of which 18,050 transcripts mapped to Gene Ontology in the tree classes (Fig. 4, Table S2) such as biological process (15,353), cellular component (15,401), and molecular function (14,354), and 2357 transcripts were identified as coding enzymes, totalling 680 different enzymes of the six classes and 16,091 transcripts coding at least one domain region in proteins (4838 different domains were identified).

Raw reads were deposited in the NCBI database and are accessible via BioProject accession number PRJNA615000 (https://www.ncbi.nlm.nih.gov/bioproject/PRJNA615000) and Sequence Read Archive (SRA) with accession number SRX7990430 (https://www.ncbi.nlm.nih.gov/sra/SRX7990430). Additionally, transcriptome shotgun assembly sequences and functional annotations are available via Discover Mendeley Data (https://data.mendeley.com/datasets/2csj3h29fr/1).

## 2 Experimental design, materials, and methods

### 2.1 Plant materials

One hundred ripe fruits (90 days after anthesis) were randomly collected from the accession code PER1000425 from the *M. dubia* germplasm collection (03°57′17′' S, 73°24′55′' W) at the Instituto Nacional de Innovación Agraria (INIA) of Peru, Region Loreto. Seeds were extracted from ripe fruits and cleaned from the pulp by washing in running water and rinsed in sterilized ultrapure water. Further, seeds were imbibited during 24 h, transferred between paper towels moistened with sterilized ultrapure water and then germinated for one week under dark conditions at 25 °C and 95% relative humidity. Next, germinated seeds were transplanted and grown under hydroponic conditions for one month with culture conditions of 25 °C, 12 h light-dark photoperiod cycle with 100 μmol photons.m^2^.*s* ^−^ ^1^ of light intensity, and 95% of relative humidity in a climatic chamber (Climacell^Ⓡ^ EVO 404, München, Germany). Plant material was harvested in triplicate in several steps: 1) imbibited seeds, 2) germinated seeds, and 3) seedlings at four growth periods (week 1, 2, 3, and 4). Obtained samples were immediately stored at −80 °C until further use. A graphical representation of the workflow is provided in the Supplementary material (Fig. S1).

### 2.2 Total RNA isolation, library preparation and next-generation DNA sequencing

Total RNA was isolated following the manufacturer's instructions using the RNeasy Plant Mini Kit (Qiagen, Hilden, Germany). The quantity and quality of total RNA were determined by spectrophotometric analysis with a Nanodrop 2000 Spectrophotometer and RNA integrity using a 2100 Bioanalyzer (Agilent, CA, USA). Total RNA from each type of plant material (i.e., imbibited seeds, germinated seeds, and seedlings of one, two, three, and four weeks old) were pooled in equimolar ratios to construct the cDNA library. The cDNA library with 500 bp size was constructed following the manufacturer's instructions using the TruSeq Stranded mRNA Sample Preparation Kit (Illumina, San Diego, USA). The cDNA library was quantified using the Qubit™ dsDNA HS Assay Kit (Thermo Fisher Scientific, Waltham, USA) and paired-end sequenced (2 × 150 bp) on an Illumina HiSeq™2500 platform.

### 2.3 De novo assembly and functional annotation

Raw paired-end sequences were uploaded as FASTQ files to Galaxy (https://usegalaxy.org/) and Kbase (http://kbase.us/) bioinformatic platforms. In these bioinformatic platforms the quality of the raw data was assessed using FastQC [3] and pre-processed with Trimommatic [4] to trim off adaptor sequences, low quality bases (≤ Q20) and short sequences (≤ 50 bp in length). The remaining high quality reads were de novo assembled using Trinity v2.9.1 [5] with default parameters and a minimum contig length of 500 bp. Additionally, multiple transcripts of genes were combined into a single sequence with SuperTranscripts v2.9.1 [6]. The completeness of assembled transcripts was evaluated using the Benchmarking Universal Single-Copy Orthologs (BUSCO) [7] software v2/v3 as implemented in the web-based server gVolante [8].

Futhermore, the assembled transcriptome was functionally annotated with the following software tools: 1) TransDecoder v3.0.1 [9] was used to predict Open Reading Frames (ORFs) and to obtain protein sequences of at least 100 amino acids in length; 2) Kyoto Encyclopedia of Genes and Genomes (KEGG) Automatic Annotation Server (KAAS) v2.1 (https://www.genome.jp/tools/kaas/) with default threshold bit-score value of 60, single-directional best hit (SBH) method, BLASTx program, and genes dataset of ten eudicots (*Arabidopsis thaliana, Brassica rapa, Citrus sinensis, Eucalyptus grandis, Populus trichocarpa, Rosa chinensis, Solanum lycopersicum, Tarenaya hassleriana, Theobroma cacao*, and *Vitis vinifera*) was used to assign KEGG Orthology IDs, to obtain BRITE hierarchies, and to generate the metabolic pathway maps; and 3) FunctionAnnotator (http://fa.cgu.edu.tw/index.php) was used with default parameters with the following analysis module: Best hit in NCBI non-redundant protein database (Taxonomic distribution and GO function annotation [Blast2GO]), Enzyme prediction (PRIAM database), and Domain region identification (Domain finder).

## Declaration of Competing Interest

The authors declare that they have no known competing financial interests or personal relationships which have, or could be perceived to have, influenced the work reported in this article.

## Appendix Supplementary materials

Image, application 1

Image, application 2
