# Supplementary material for: Dataset of de novo assembly and functional annotation of the transcriptome during germination and initial growth of seedlings of Myrciaria Dubia “camu-camu”
Source: Data Brief. 2020 Jun 11;31:105834. doi: 10.1016/j.dib.2020.105834 (PMC7305401; doi:10.1016/j.dib.2020.105834)
Supplement: Supplementary file 1 [file mmc1.docx]

**Supplementary material**


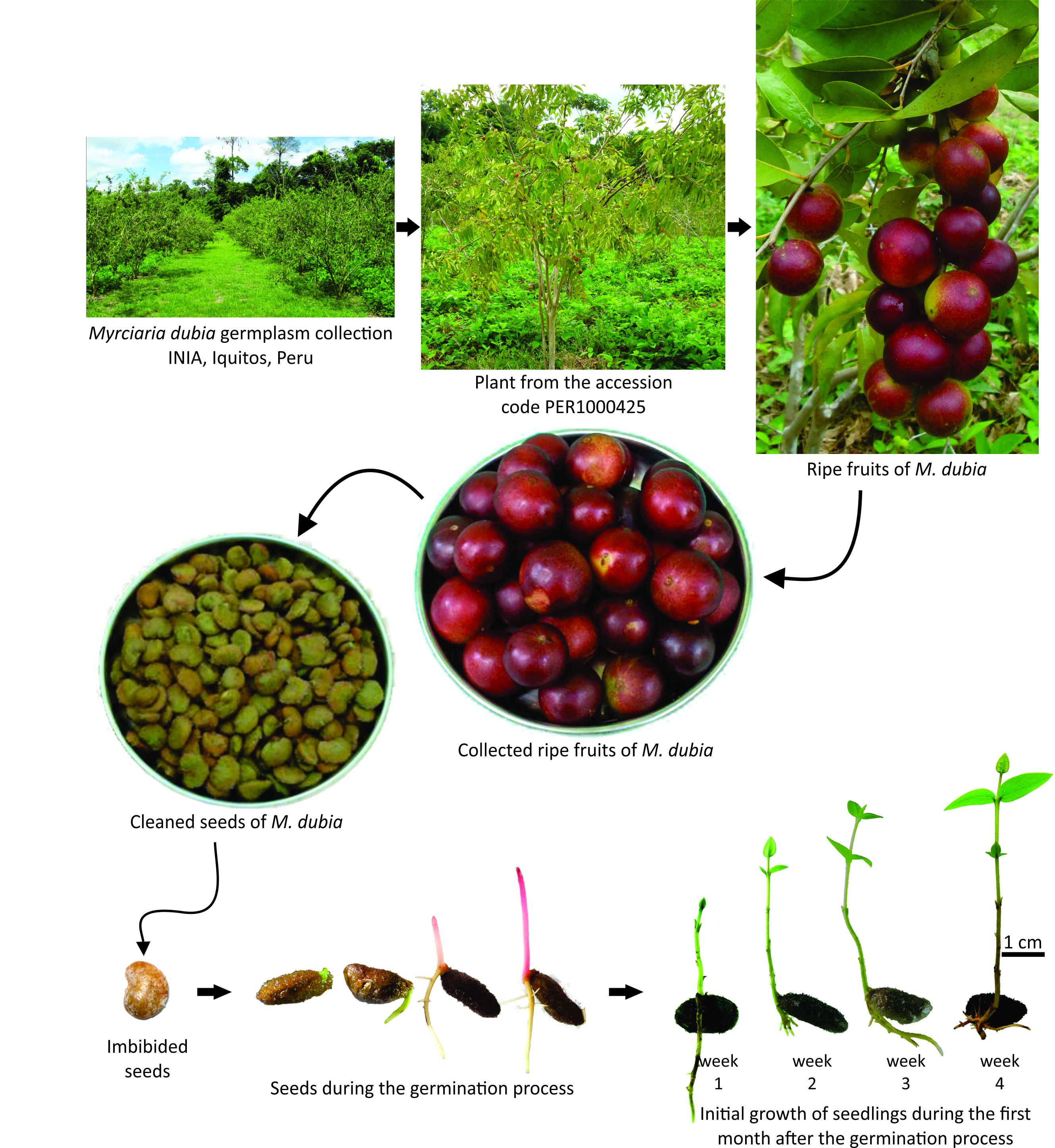


Fig. S1. Flow diagram of methodological approaches

Table S1. List of metabolic pathways mapped from the de novo assembled transcripts of the transcriptome obtained during germination and initial growth of seedlings of *M. dubia*

| **KEGG Pathway ID** | **Metabolic Pathway** | **N° Enzymes/Proteins Mapped** |
| --- | --- | --- |
| 03010 | Ribosome | 117 |
| 03040 | Spliceosome | 102 |
| 03013 | RNA transport | 94 |
| 04141 | Protein processing in endoplasmic reticulum | 76 |
| 04714 | Thermogenesis | 74 |
| 00190 | Oxidative phosphorylation | 65 |
| 04120 | Ubiquitin mediated proteolysis | 57 |
| 04144 | Endocytosis | 57 |
| 03008 | Ribosome biogenesis in eukaryotes | 54 |
| 04110 | Cell cycle | 50 |
| 03018 | RNA degradation | 49 |
| 03015 | mRNA surveillance pathway | 48 |
| 00230 | Purine metabolism | 46 |
| 00270 | Cysteine and methionine metabolism | 42 |
| 00520 | Amino sugar and nucleotide sugar metabolism | 41 |
| 04075 | Plant hormone signal transduction | 41 |
| 00564 | Glycerophospholipid metabolism | 39 |
| 04016 | MAPK signaling pathway - plant | 39 |
| 04140 | Autophagy - animal | 38 |
| 04142 | Lysosome | 38 |
| 04146 | Peroxisome | 38 |
| 00010 | Glycolysis / Gluconeogenesis | 35 |
| 03050 | Proteasome | 35 |
| 03420 | Nucleotide excision repair | 35 |
| 05200 | Pathways in cancer | 35 |
| 00260 | Glycine, serine and threonine metabolism | 34 |
| 00620 | Pyruvate metabolism | 33 |
| 00860 | Porphyrin and chlorophyll metabolism | 32 |
| 04626 | Plant-pathogen interaction | 32 |
| 00630 | Glyoxylate and dicarboxylate metabolism | 31 |
| 00500 | Starch and sucrose metabolism | 30 |
| 00510 | N-Glycan biosynthesis | 30 |
| 00561 | Glycerolipid metabolism | 30 |
| 00900 | Terpenoid backbone biosynthesis | 30 |
| 03022 | Basal transcription factors | 29 |
| 04114 | Oocyte meiosis | 29 |
| 04145 | Phagosome | 29 |
| 04150 | mTOR signaling pathway | 29 |
| 00240 | Pyrimidine metabolism | 28 |
| 00250 | Alanine, aspartate and glutamate metabolism | 28 |
| 00970 | Aminoacyl-tRNA biosynthesis | 27 |
| 03020 | RNA polymerase | 27 |
| 03030 | DNA replication | 27 |
| 00195 | Photosynthesis | 26 |
| 00710 | Carbon fixation in photosynthetic organisms | 26 |
| 03060 | Protein export | 26 |
| 04218 | Cellular senescence | 26 |
| 04723 | Retrograde endocannabinoid signaling | 26 |
| 00562 | Inositol phosphate metabolism | 25 |
| 04151 | PI3K-Akt signaling pathway | 25 |
| 03440 | Homologous recombination | 24 |
| 04152 | AMPK signaling pathway | 24 |
| 00280 | Valine, leucine and isoleucine degradation | 23 |
| 00330 | Arginine and proline metabolism | 23 |
| 00400 | Phenylalanine, tyrosine and tryptophan biosynthesis | 23 |
| 00513 | Various types of N-glycan biosynthesis | 23 |
| 04136 | Autophagy - other | 23 |
| 03410 | Base excision repair | 22 |
| 00130 | Ubiquinone and other terpenoid-quinone biosynthesis | 21 |
| 00220 | Arginine biosynthesis | 21 |
| 00563 | Glycosylphosphatidylinositol (GPI)-anchor | 21 |
| 00640 | Propanoate metabolism | 21 |
| 04070 | Phosphatidylinositol signaling system | 21 |
| 04217 | Necroptosis | 21 |
| 00020 | Citrate cycle (TCA cycle) | 20 |
| 00051 | Fructose and mannose metabolism | 20 |
| 04066 | HIF-1 signaling pathway | 20 |
| 04712 | Circadian rhythm - plant | 20 |
| 04810 | Regulation of actin cytoskeleton | 20 |
| 00350 | Tyrosine metabolism | 19 |
| 00680 | Methane metabolism | 19 |
| 00940 | Phenylpropanoid biosynthesis | 19 |
| 03430 | Mismatch repair | 19 |
| 04071 | Sphingolipid signaling pathway | 19 |
| 00030 | Pentose phosphate pathway | 18 |
| 00053 | Ascorbate and aldarate metabolism | 18 |
| 00480 | Glutathione metabolism | 18 |
| 00790 | Folate biosynthesis | 18 |
| 00906 | Carotenoid biosynthesis | 18 |
| 00983 | Drug metabolism - other enzymes | 18 |
| 04130 | SNARE interactions in vesicular transport | 18 |
| 00061 | Fatty acid biosynthesis | 17 |
| 00100 | Steroid biosynthesis | 17 |
| 00410 | beta-Alanine metabolism | 17 |
| 04310 | Wnt signaling pathway | 17 |
| 00052 | Galactose metabolism | 16 |
| 00380 | Tryptophan metabolism | 16 |
| 00941 | Flavonoid biosynthesis | 16 |
| 04011 | MAPK signaling pathway - yeast | 16 |
| 04623 | Cytosolic DNA-sensing pathway | 16 |
| 04919 | Thyroid hormone signaling pathway | 16 |
| 00310 | Lysine degradation | 15 |
| 00360 | Phenylalanine metabolism | 15 |
| 00770 | Pantothenate and CoA biosynthesis | 15 |
| 00920 | Sulfur metabolism | 15 |
| 04530 | Tight junction | 15 |
| 00040 | Pentose and glucuronate interconversions | 14 |
| 00071 | Fatty acid degradation | 14 |
| 00600 | Sphingolipid metabolism | 14 |
| 00720 | Carbon fixation pathways in prokaryotes | 14 |
| 00760 | Nicotinate and nicotinamide metabolism | 14 |
| 04210 | Apoptosis | 14 |
| 00592 | alpha-Linolenic acid metabolism | 13 |
| 00650 | Butanoate metabolism | 13 |
| 00340 | Histidine metabolism | 12 |
| 02020 | Two-component system | 12 |
| 02024 | Quorum sensing | 12 |
| 04010 | MAPK signaling pathway | 12 |
| 04072 | Phospholipase D signaling pathway | 12 |
| 04115 | p53 signaling pathway | 12 |
| 04213 | Longevity regulating pathway - multiple species | 12 |
| 00196 | Photosynthesis - antenna proteins | 11 |
| 00730 | Thiamine metabolism | 11 |
| 00910 | Nitrogen metabolism | 11 |
| 04014 | Ras signaling pathway | 11 |
| 00290 | Valine, leucine and isoleucine biosynthesis | 10 |
| 00450 | Selenocompound metabolism | 10 |
| 00670 | One carbon pool by folate | 10 |
| 00740 | Riboflavin metabolism | 10 |
| 04024 | cAMP signaling pathway | 10 |
| 00460 | Cyanoamino acid metabolism | 9 |
| 00511 | Other glycan degradation | 9 |
| 00750 | Vitamin B6 metabolism | 9 |
| 00960 | Tropane, piperidine and pyridine alkaloid biosynthesis | 9 |
| 01040 | Biosynthesis of unsaturated fatty acids | 9 |
| 02010 | ABC transporters | 9 |
| 04022 | cGMP-PKG signaling pathway | 9 |
| 04330 | Notch signaling pathway | 9 |
| 04510 | Focal adhesion | 9 |
| 00073 | Cutin, suberine and wax biosynthesis | 8 |
| 00590 | Arachidonic acid metabolism | 8 |
| 00780 | Biotin metabolism | 8 |
| 00950 | Isoquinoline alkaloid biosynthesis | 8 |
| 04020 | Calcium signaling pathway | 8 |
| 04390 | Hippo signaling pathway | 8 |
| 00062 | Fatty acid elongation | 7 |
| 00300 | Lysine biosynthesis | 7 |
| 00565 | Ether lipid metabolism | 7 |
| 00905 | Brassinosteroid biosynthesis | 7 |
| 03450 | Non-homologous end-joining | 7 |
| 04122 | Sulfur relay system | 7 |
| 04216 | Ferroptosis | 7 |
| 04520 | Adherens junction | 7 |
| 04710 | Circadian rhythm | 7 |
| 00514 | Other types of O-glycan biosynthesis | 6 |
| 00540 | Lipopolysaccharide biosynthesis | 6 |
| 00904 | Diterpenoid biosynthesis | 6 |
| 00909 | Sesquiterpenoid and triterpenoid biosynthesis | 6 |
| 04015 | Rap1 signaling pathway | 6 |
| 04540 | Gap junction | 6 |
| 00660 | C5-Branched dibasic acid metabolism | 5 |
| 00980 | Metabolism of xenobiotics by cytochrome P450 | 5 |
| 00591 | Linoleic acid metabolism | 4 |
| 00908 | Zeatin biosynthesis | 4 |
| 00945 | Stilbenoid, diarylheptanoid and gingerol biosynthesis | 4 |
| 00982 | Drug metabolism - cytochrome P450 | 4 |
| 04550 | Signaling pathways regulating pluripotency of stem cells | 4 |
| 00785 | Lipoic acid metabolism | 3 |
| 00944 | Flavone and flavonol biosynthesis | 3 |
| 00901 | Indole alkaloid biosynthesis | 1 |
| 00943 | Isoflavonoid biosynthesis | 1 |

| **Gene Onthology (GO) ID** | **Biological Process** | **Number of Transcripts** |
| --- | --- | --- |
| GO:0008152 | metabolic process | 631 |
| GO:0006468 | protein phosphorylation | 626 |
| GO:0055114 | oxidation-reduction process | 520 |
| GO:0006355 | regulation of transcription, DNA-dependent | 363 |
| GO:0045893 | positive regulation of transcription, DNA-dependent | 305 |
| GO:0006952 | defense response | 297 |
| GO:0006508 | proteolysis | 244 |
| GO:0006412 | translation | 233 |
| GO:0055085 | transmembrane transport | 211 |
| GO:0005975 | carbohydrate metabolic process | 210 |
| GO:0046777 | protein autophosphorylation | 189 |
| GO:0006979 | response to oxidative stress | 180 |
| GO:0006457 | protein folding | 167 |
| GO:0009414 | response to water deprivation | 139 |
| GO:0006278 | RNA-dependent DNA replication | 124 |
| GO:0016192 | vesicle-mediated transport | 123 |
| GO:0006629 | lipid metabolic process | 122 |
| GO:0009651 | response to salt stress | 122 |
| GO:0032259 | methylation | 114 |
| GO:0006511 | ubiquitin-dependent protein catabolic process | 112 |
| GO:0031348 | negative regulation of defense response | 109 |
| GO:0016310 | phosphorylation | 108 |
| GO:0006200 | ATP catabolic process | 105 |
| GO:0046686 | response to cadmium ion | 104 |
| GO:0090305 | nucleic acid phosphodiester bond hydrolysis | 95 |
| GO:0006950 | response to stress | 91 |
| GO:0009793 | embryo development ending in seed dormancy | 87 |
| GO:0016311 | dephosphorylation | 84 |
| GO:0042742 | defense response to bacterium | 83 |
| GO:0045454 | cell redox homeostasis | 81 |
| GO:0006810 | transport | 80 |
| GO:0006413 | translational initiation | 79 |
| GO:0009611 | response to wounding | 79 |
| GO:0016567 | protein ubiquitination | 78 |
| GO:0006470 | protein dephosphorylation | 69 |
| GO:0009733 | response to auxin stimulus | 67 |
| GO:0009409 | response to cold | 62 |
| GO:0006623 | protein targeting to vacuole | 60 |
| GO:0006633 | fatty acid biosynthetic process | 60 |
| GO:0006098 | pentose-phosphate shunt | 57 |
| GO:0048364 | root development | 57 |
| GO:0000278 | mitotic cell cycle | 53 |
| GO:0010200 | response to chitin | 50 |
| GO:0010413 | glucuronoxylan metabolic process | 50 |
| GO:0080167 | response to karrikin | 48 |
| GO:0009630 | gravitropism | 47 |
| GO:0009751 | response to salicylic acid stimulus | 47 |
| GO:0048767 | root hair elongation | 45 |
| GO:0009624 | response to nematode | 43 |
| GO:0009737 | response to abscisic acid stimulus | 43 |
| GO:0006886 | intracellular protein transport | 42 |
| GO:0009750 | response to fructose stimulus | 42 |
| GO:0000911 | cytokinesis by cell plate formation | 41 |
| GO:0001510 | RNA methylation | 41 |
| GO:0018108 | peptidyl-tyrosine phosphorylation | 40 |
| GO:0007264 | small GTPase mediated signal transduction | 39 |
| GO:0050832 | defense response to fungus | 39 |
| GO:0035556 | intracellular signal transduction | 38 |
| GO:0006351 | transcription, DNA-dependent | 37 |
| GO:0016558 | protein import into peroxisome matrix | 37 |
| GO:0071472 | cellular response to salt stress | 37 |
| GO:0010048 | vernalization response | 35 |
| GO:0010075 | regulation of meristem growth | 35 |
| GO:0030001 | metal ion transport | 35 |
| GO:0006486 | protein glycosylation | 34 |
| GO:0006499 | N-terminal protein myristoylation | 34 |
| GO:0030244 | cellulose biosynthetic process | 34 |
| GO:0048316 | seed development | 34 |
| GO:0009873 | ethylene mediated signaling pathway | 33 |
| GO:0001666 | response to hypoxia | 32 |
| GO:0006414 | translational elongation | 32 |
| GO:0009658 | chloroplast organization | 32 |
| GO:0010286 | heat acclimation | 32 |
| GO:0016132 | brassinosteroid biosynthetic process | 32 |
| GO:0048573 | photoperiodism, flowering | 32 |
| GO:0006184 | GTP catabolic process | 31 |
| GO:0007018 | microtubule-based movement | 31 |
| GO:0009627 | systemic acquired resistance | 31 |
| GO:0015979 | photosynthesis | 31 |
| GO:0045492 | xylan biosynthetic process | 31 |
| GO:0000398 | nuclear mRNA splicing, via spliceosome | 30 |
| GO:0007165 | signal transduction | 30 |
| GO:0009560 | embryo sac egg cell differentiation | 30 |
| GO:0009908 | flower development | 30 |
| GO:0010363 | regulation of plant-type hypersensitive response | 30 |
| GO:0015031 | protein transport | 30 |
| GO:0016036 | cellular response to phosphate starvation | 30 |
| GO:0009615 | response to virus | 29 |
| GO:0042538 | hyperosmotic salinity response | 29 |
| GO:0006855 | drug transmembrane transport | 28 |
| GO:0007623 | circadian rhythm | 28 |
| GO:0009058 | biosynthetic process | 28 |
| GO:0009825 | multidimensional cell growth | 28 |
| GO:0010162 | seed dormancy | 28 |
| GO:0032508 | DNA duplex unwinding | 28 |
| GO:0048193 | Golgi vesicle transport | 28 |
| GO:0000160 | two-component signal transduction system (phosphorelay) | 27 |
| GO:0006364 | rRNA processing | 27 |
| GO:0009790 | embryo development | 27 |
| GO:0006397 | mRNA processing | 26 |
| GO:0006661 | phosphatidylinositol biosynthetic process | 26 |
| GO:0009555 | pollen development | 26 |
| GO:0010103 | stomatal complex morphogenesis | 26 |
| GO:0010224 | response to UV-B | 26 |
| GO:0032851 | positive regulation of Rab GTPase activity | 26 |
| GO:0006396 | RNA processing | 25 |
| GO:0006869 | lipid transport | 25 |
| GO:0009734 | auxin mediated signaling pathway | 25 |
| GO:0010029 | regulation of seed germination | 25 |
| GO:0010264 | myo-inositol hexakisphosphate biosynthetic process | 25 |
| GO:0034976 | response to endoplasmic reticulum stress | 25 |
| GO:0006281 | DNA repair | 24 |
| GO:0006346 | methylation-dependent chromatin silencing | 24 |
| GO:0009753 | response to jasmonic acid stimulus | 24 |
| GO:0016226 | iron-sulfur cluster assembly | 24 |
| GO:0034968 | histone lysine methylation | 24 |
| GO:0003333 | amino acid transmembrane transport | 23 |
| GO:0006007 | glucose catabolic process | 23 |
| GO:0006357 | regulation of transcription from RNA polymerase II promoter | 23 |
| GO:0006635 | fatty acid beta-oxidation | 23 |
| GO:0009664 | plant-type cell wall organization | 23 |
| GO:0009813 | flavonoid biosynthetic process | 23 |
| GO:0015706 | nitrate transport | 23 |
| GO:0070588 | calcium ion transmembrane transport | 23 |
| GO:0000226 | microtubule cytoskeleton organization | 22 |
| GO:0006096 | glycolysis | 22 |
| GO:0006260 | DNA replication | 22 |
| GO:0006289 | nucleotide-excision repair | 22 |
| GO:0009294 | DNA mediated transformation | 22 |
| GO:0009845 | seed germination | 22 |
| GO:0009860 | pollen tube growth | 22 |
| GO:0042545 | cell wall modification | 22 |
| GO:0048868 | pollen tube development | 22 |
| GO:0051788 | response to misfolded protein | 22 |
| GO:0006284 | base-excision repair | 21 |
| GO:0006310 | DNA recombination | 21 |
| GO:0009607 | response to biotic stimulus | 21 |
| GO:0009646 | response to absence of light | 21 |
| GO:0010584 | pollen exine formation | 21 |
| GO:0022900 | electron transport chain | 21 |
| GO:0071805 | potassium ion transmembrane transport | 21 |
| GO:0007017 | microtubule-based process | 20 |
| GO:0009165 | nucleotide biosynthetic process | 20 |
| GO:0009644 | response to high light intensity | 20 |
| GO:0009744 | response to sucrose stimulus | 20 |
| GO:0015693 | magnesium ion transport | 20 |
| GO:0016556 | mRNA modification | 20 |
| GO:0019375 | galactolipid biosynthetic process | 20 |
| GO:0042744 | hydrogen peroxide catabolic process | 20 |
| GO:0006857 | oligopeptide transport | 19 |
| GO:0009735 | response to cytokinin stimulus | 19 |
| GO:0015996 | chlorophyll catabolic process | 19 |
| GO:0006094 | gluconeogenesis | 18 |
| GO:0006261 | DNA-dependent DNA replication | 18 |
| GO:0006606 | protein import into nucleus | 18 |
| GO:0009086 | methionine biosynthetic process | 18 |
| GO:0009620 | response to fungus | 18 |
| GO:0010332 | response to gamma radiation | 18 |
| GO:0010417 | glucuronoxylan biosynthetic process | 18 |
| GO:0019243 | methylglyoxal catabolic process to D-lactate | 18 |
| GO:0035335 | peptidyl-tyrosine dephosphorylation | 18 |
| GO:0042254 | ribosome biogenesis | 18 |
| GO:0000956 | nuclear-transcribed mRNA catabolic process | 17 |
| GO:0006073 | cellular glucan metabolic process | 17 |
| GO:0009934 | regulation of meristem structural organization | 17 |
| GO:0010193 | response to ozone | 17 |
| GO:0010468 | regulation of gene expression | 17 |
| GO:0010540 | basipetal auxin transport | 17 |
| GO:0015074 | DNA integration | 17 |
| GO:0071281 | cellular response to iron ion | 17 |
| GO:0071456 | cellular response to hypoxia | 17 |
| GO:0001731 | formation of translation preinitiation complex | 16 |
| GO:0007030 | Golgi organization | 16 |
| GO:0008380 | RNA splicing | 16 |
| GO:0009407 | toxin catabolic process | 16 |
| GO:0009944 | polarity specification of adaxial/abaxial axis | 16 |
| GO:0010150 | leaf senescence | 16 |
| GO:0015991 | ATP hydrolysis coupled proton transport | 16 |
| GO:0016049 | cell growth | 16 |
| GO:0016051 | carbohydrate biosynthetic process | 16 |
| GO:0016117 | carotenoid biosynthetic process | 16 |
| GO:0016998 | cell wall macromolecule catabolic process | 16 |
| GO:0031347 | regulation of defense response | 16 |
| GO:0045332 | phospholipid translocation | 16 |
| GO:0046520 | sphingoid biosynthetic process | 16 |
| GO:0051301 | cell division | 16 |
| GO:0051607 | defense response to virus | 16 |
| GO:0000394 | RNA splicing, via endonucleolytic cleavage and ligation | 15 |
| GO:0001522 | pseudouridine synthesis | 15 |
| GO:0009827 | plant-type cell wall modification | 15 |
| GO:0009833 | primary cell wall biogenesis | 15 |
| GO:0010118 | stomatal movement | 15 |
| GO:0045489 | pectin biosynthetic process | 15 |
| GO:0007275 | multicellular organismal development | 14 |
| GO:0009416 | response to light stimulus | 14 |
| GO:0009684 | indoleacetic acid biosynthetic process | 14 |
| GO:0009739 | response to gibberellin stimulus | 14 |
| GO:0009958 | positive gravitropism | 14 |
| GO:0010440 | stomatal lineage progression | 14 |
| GO:0019252 | starch biosynthetic process | 14 |
| GO:0048510 | regulation of timing of transition from vegetative to reproductive phase | 14 |
| GO:0048768 | root hair cell tip growth | 14 |
| GO:0051707 | response to other organism | 14 |
| GO:0006071 | glycerol metabolic process | 13 |
| GO:0006301 | postreplication repair | 13 |
| GO:0006631 | fatty acid metabolic process | 13 |
| GO:0009723 | response to ethylene stimulus | 13 |
| GO:0009741 | response to brassinosteroid stimulus | 13 |
| GO:0009765 | photosynthesis, light harvesting | 13 |
| GO:0009853 | photorespiration | 13 |
| GO:0009910 | negative regulation of flower development | 13 |
| GO:0009932 | cell tip growth | 13 |
| GO:0010119 | regulation of stomatal movement | 13 |
| GO:0016926 | protein desumoylation | 13 |
| GO:0019509 | L-methionine salvage from methylthioadenosine | 13 |
| GO:0030488 | tRNA methylation | 13 |
| GO:0031047 | gene silencing by RNA | 13 |
| GO:0043247 | telomere maintenance in response to DNA damage | 13 |
| GO:0044070 | regulation of anion transport | 13 |
| GO:0046854 | phosphatidylinositol phosphorylation | 13 |
| GO:0046939 | nucleotide phosphorylation | 13 |
| GO:0000266 | mitochondrial fission | 12 |
| GO:0005982 | starch metabolic process | 12 |
| GO:0006012 | galactose metabolic process | 12 |
| GO:0006334 | nucleosome assembly | 12 |
| GO:0006626 | protein targeting to mitochondrion | 12 |
| GO:0006904 | vesicle docking involved in exocytosis | 12 |
| GO:0009553 | embryo sac development | 12 |
| GO:0009639 | response to red or far red light | 12 |
| GO:0009648 | photoperiodism | 12 |
| GO:0009789 | positive regulation of abscisic acid mediated signaling pathway | 12 |
| GO:0009867 | jasmonic acid mediated signaling pathway | 12 |
| GO:0009911 | positive regulation of flower development | 12 |
| GO:0009965 | leaf morphogenesis | 12 |
| GO:0010207 | photosystem II assembly | 12 |
| GO:0010267 | production of ta-siRNAs involved in RNA interference | 12 |
| GO:0043086 | negative regulation of catalytic activity | 12 |
| GO:0043248 | proteasome assembly | 12 |
| GO:0046482 | para-aminobenzoic acid metabolic process | 12 |
| GO:0051258 | protein polymerization | 12 |
| GO:0000038 | very long chain fatty acid metabolic process | 11 |
| GO:0006662 | glycerol ether metabolic process | 11 |
| GO:0006914 | autophagy | 11 |
| GO:0007010 | cytoskeleton organization | 11 |
| GO:0007568 | aging | 11 |
| GO:0008284 | positive regulation of cell proliferation | 11 |
| GO:0009640 | photomorphogenesis | 11 |
| GO:0009693 | ethylene biosynthetic process | 11 |
| GO:0009902 | chloroplast relocation | 11 |
| GO:0009960 | endosperm development | 11 |
| GO:0010053 | root epidermal cell differentiation | 11 |
| GO:0010090 | trichome morphogenesis | 11 |
| GO:0010161 | red light signaling pathway | 11 |
| GO:0010431 | seed maturation | 11 |
| GO:0015995 | chlorophyll biosynthetic process | 11 |
| GO:0030026 | cellular manganese ion homeostasis | 11 |
| GO:0031167 | rRNA methylation | 11 |
| GO:0035194 | posttranscriptional gene silencing by RNA | 11 |
| GO:0048440 | carpel development | 11 |
| GO:0006066 | alcohol metabolic process | 10 |
| GO:0006415 | translational termination | 10 |
| GO:0006541 | glutamine metabolic process | 10 |
| GO:0006833 | water transport | 10 |
| GO:0007049 | cell cycle | 10 |
| GO:0007067 | mitosis | 10 |
| GO:0009561 | megagametogenesis | 10 |
| GO:0009736 | cytokinin mediated signaling pathway | 10 |
| GO:0009805 | coumarin biosynthetic process | 10 |
| GO:0009846 | pollen germination | 10 |
| GO:0009862 | systemic acquired resistance, salicylic acid mediated signaling pathway | 10 |
| GO:0009880 | embryonic pattern specification | 10 |
| GO:0010025 | wax biosynthetic process | 10 |
| GO:0010026 | trichome differentiation | 10 |
| GO:0010027 | thylakoid membrane organization | 10 |
| GO:0010102 | lateral root morphogenesis | 10 |
| GO:0010114 | response to red light | 10 |
| GO:0043087 | regulation of GTPase activity | 10 |
| GO:0046688 | response to copper ion | 10 |
| GO:0046855 | inositol phosphate dephosphorylation | 10 |
| GO:0048268 | clathrin coat assembly | 10 |
| GO:0048544 | recognition of pollen | 10 |
| GO:0051645 | Golgi localization | 10 |
| GO:0000079 | regulation of cyclin-dependent protein kinase activity | 9 |
| GO:0000184 | nuclear-transcribed mRNA catabolic process, nonsense-mediated decay | 9 |
| GO:0006099 | tricarboxylic acid cycle | 9 |
| GO:0006376 | mRNA splice site selection | 9 |
| GO:0006655 | phosphatidylglycerol biosynthetic process | 9 |
| GO:0006694 | steroid biosynthetic process | 9 |
| GO:0006887 | exocytosis | 9 |
| GO:0007155 | cell adhesion | 9 |
| GO:0007205 | activation of protein kinase C activity by G-protein coupled receptor protein signaling pathway | 9 |
| GO:0009408 | response to heat | 9 |
| GO:0009411 | response to UV | 9 |
| GO:0009631 | cold acclimation | 9 |
| GO:0009638 | phototropism | 9 |
| GO:0009695 | jasmonic acid biosynthetic process | 9 |
| GO:0009809 | lignin biosynthetic process | 9 |
| GO:0009851 | auxin biosynthetic process | 9 |
| GO:0010020 | chloroplast fission | 9 |
| GO:0010197 | polar nucleus fusion | 9 |
| GO:0030042 | actin filament depolymerization | 9 |
| GO:0035434 | copper ion transmembrane transport | 9 |
| GO:0042127 | regulation of cell proliferation | 9 |
| GO:0042546 | cell wall biogenesis | 9 |
| GO:0042787 | protein ubiquitination involved in ubiquitin-dependent protein catabolic process | 9 |
| GO:0042939 | tripeptide transport | 9 |
| GO:0043622 | cortical microtubule organization | 9 |
| GO:0044237 | cellular metabolic process | 9 |
| GO:0046283 | anthocyanin metabolic process | 9 |
| GO:0048367 | shoot development | 9 |
| GO:0048831 | regulation of shoot development | 9 |
| GO:0051302 | regulation of cell division | 9 |
| GO:0051510 | regulation of unidimensional cell growth | 9 |
| GO:0006465 | signal peptide processing | 8 |
| GO:0006487 | protein N-linked glycosylation | 8 |
| GO:0006506 | GPI anchor biosynthetic process | 8 |
| GO:0006544 | glycine metabolic process | 8 |
| GO:0006555 | methionine metabolic process | 8 |
| GO:0006680 | glucosylceramide catabolic process | 8 |
| GO:0006825 | copper ion transport | 8 |
| GO:0006865 | amino acid transport | 8 |
| GO:0008202 | steroid metabolic process | 8 |
| GO:0008299 | isoprenoid biosynthetic process | 8 |
| GO:0009089 | lysine biosynthetic process via diaminopimelate | 8 |
| GO:0009231 | riboflavin biosynthetic process | 8 |
| GO:0009690 | cytokinin metabolic process | 8 |
| GO:0009697 | salicylic acid biosynthetic process | 8 |
| GO:0009826 | unidimensional cell growth | 8 |
| GO:0010054 | trichoblast differentiation | 8 |
| GO:0010155 | regulation of proton transport | 8 |
| GO:0015700 | arsenite transport | 8 |
| GO:0015721 | bile acid and bile salt transport | 8 |
| GO:0015786 | UDP-glucose transport | 8 |
| GO:0015937 | coenzyme A biosynthetic process | 8 |
| GO:0015986 | ATP synthesis coupled proton transport | 8 |
| GO:0016114 | terpenoid biosynthetic process | 8 |
| GO:0016925 | protein sumoylation | 8 |
| GO:0019748 | secondary metabolic process | 8 |
| GO:0031087 | deadenylation-independent decapping of nuclear-transcribed mRNA | 8 |
| GO:0031425 | chloroplast RNA processing | 8 |
| GO:0034220 | ion transmembrane transport | 8 |
| GO:0040007 | growth | 8 |
| GO:0045927 | positive regulation of growth | 8 |
| GO:0046835 | carbohydrate phosphorylation | 8 |
| GO:0048281 | inflorescence morphogenesis | 8 |
| GO:0048838 | release of seed from dormancy | 8 |
| GO:0050826 | response to freezing | 8 |
| GO:0051017 | actin filament bundle assembly | 8 |
| GO:0055075 | potassium ion homeostasis | 8 |
| GO:0000373 | Group II intron splicing | 7 |
| GO:0005978 | glycogen biosynthetic process | 7 |
| GO:0006164 | purine nucleotide biosynthetic process | 7 |
| GO:0006313 | transposition, DNA-mediated | 7 |
| GO:0006464 | protein modification process | 7 |
| GO:0006571 | tyrosine biosynthetic process | 7 |
| GO:0006605 | protein targeting | 7 |
| GO:0006744 | ubiquinone biosynthetic process | 7 |
| GO:0006777 | Mo-molybdopterin cofactor biosynthetic process | 7 |
| GO:0006801 | superoxide metabolic process | 7 |
| GO:0006807 | nitrogen compound metabolic process | 7 |
| GO:0006813 | potassium ion transport | 7 |
| GO:0006814 | sodium ion transport | 7 |
| GO:0006891 | intra-Golgi vesicle-mediated transport | 7 |
| GO:0007015 | actin filament organization | 7 |
| GO:0007020 | microtubule nucleation | 7 |
| GO:0007267 | cell-cell signaling | 7 |
| GO:0008283 | cell proliferation | 7 |
| GO:0008643 | carbohydrate transport | 7 |
| GO:0008654 | phospholipid biosynthetic process | 7 |
| GO:0009062 | fatty acid catabolic process | 7 |
| GO:0009116 | nucleoside metabolic process | 7 |
| GO:0009245 | lipid A biosynthetic process | 7 |
| GO:0009306 | protein secretion | 7 |
| GO:0009767 | photosynthetic electron transport chain | 7 |
| GO:0009791 | post-embryonic development | 7 |
| GO:0009864 | induced systemic resistance, jasmonic acid mediated signaling pathway | 7 |
| GO:0009888 | tissue development | 7 |
| GO:0010112 | regulation of systemic acquired resistance | 7 |
| GO:0010158 | abaxial cell fate specification | 7 |
| GO:0010182 | sugar mediated signaling pathway | 7 |
| GO:0010206 | photosystem II repair | 7 |
| GO:0010215 | cellulose microfibril organization | 7 |
| GO:0010231 | maintenance of seed dormancy | 7 |
| GO:0015691 | cadmium ion transport | 7 |
| GO:0015824 | proline transport | 7 |
| GO:0016042 | lipid catabolic process | 7 |
| GO:0016568 | chromatin modification | 7 |
| GO:0019253 | reductive pentose-phosphate cycle | 7 |
| GO:0019722 | calcium-mediated signaling | 7 |
| GO:0031048 | chromatin silencing by small RNA | 7 |
| GO:0032544 | plastid translation | 7 |
| GO:0035019 | somatic stem cell maintenance | 7 |
| GO:0042343 | indole glucosinolate metabolic process | 7 |
| GO:0043161 | proteasomal ubiquitin-dependent protein catabolic process | 7 |
| GO:0045892 | negative regulation of transcription, DNA-dependent | 7 |
| GO:0046907 | intracellular transport | 7 |
| GO:0048366 | leaf development | 7 |
| GO:0048765 | root hair cell differentiation | 7 |
| GO:0050790 | regulation of catalytic activity | 7 |
| GO:0070475 | rRNA base methylation | 7 |
| GO:0080144 | amino acid homeostasis | 7 |
| GO:0080147 | root hair cell development | 7 |
| GO:2000069 | regulation of post-embryonic root development | 7 |
| GO:0000003 | reproduction | 6 |
| GO:0000041 | transition metal ion transport | 6 |
| GO:0000103 | sulfate assimilation | 6 |
| GO:0000723 | telomere maintenance | 6 |
| GO:0000910 | cytokinesis | 6 |
| GO:0002237 | response to molecule of bacterial origin | 6 |
| GO:0002679 | respiratory burst involved in defense response | 6 |
| GO:0006014 | D-ribose metabolic process | 6 |
| GO:0006122 | mitochondrial electron transport, ubiquinol to cytochrome c | 6 |
| GO:0006165 | nucleoside diphosphate phosphorylation | 6 |
| GO:0006306 | DNA methylation | 6 |
| GO:0006333 | chromatin assembly or disassembly | 6 |
| GO:0006352 | transcription initiation, DNA-dependent | 6 |
| GO:0006353 | transcription termination, DNA-dependent | 6 |
| GO:0006354 | transcription elongation, DNA-dependent | 6 |
| GO:0006367 | transcription initiation from RNA polymerase II promoter | 6 |
| GO:0006378 | mRNA polyadenylation | 6 |
| GO:0006400 | tRNA modification | 6 |
| GO:0006427 | histidyl-tRNA aminoacylation | 6 |
| GO:0006450 | regulation of translational fidelity | 6 |
| GO:0006491 | N-glycan processing | 6 |
| GO:0006542 | glutamine biosynthetic process | 6 |
| GO:0006598 | polyamine catabolic process | 6 |
| GO:0006614 | SRP-dependent cotranslational protein targeting to membrane | 6 |
| GO:0006644 | phospholipid metabolic process | 6 |
| GO:0006888 | ER to Golgi vesicle-mediated transport | 6 |
| GO:0006913 | nucleocytoplasmic transport | 6 |
| GO:0006915 | apoptotic process | 6 |
| GO:0007034 | vacuolar transport | 6 |
| GO:0008610 | lipid biosynthetic process | 6 |
| GO:0009072 | aromatic amino acid family metabolic process | 6 |
| GO:0009088 | threonine biosynthetic process | 6 |
| GO:0009220 | pyrimidine ribonucleotide biosynthetic process | 6 |
| GO:0009269 | response to desiccation | 6 |
| GO:0009451 | RNA modification | 6 |
| GO:0009567 | double fertilization forming a zygote and endosperm | 6 |
| GO:0009617 | response to bacterium | 6 |
| GO:0009626 | plant-type hypersensitive response | 6 |
| GO:0009642 | response to light intensity | 6 |
| GO:0009773 | photosynthetic electron transport in photosystem I | 6 |
| GO:0009785 | blue light signaling pathway | 6 |
| GO:0009816 | defense response to bacterium, incompatible interaction | 6 |
| GO:0009817 | defense response to fungus, incompatible interaction | 6 |
| GO:0009834 | secondary cell wall biogenesis | 6 |
| GO:0009863 | salicylic acid mediated signaling pathway | 6 |
| GO:0009909 | regulation of flower development | 6 |
| GO:0010208 | pollen wall assembly | 6 |
| GO:0010228 | vegetative to reproductive phase transition of meristem | 6 |
| GO:0010256 | endomembrane system organization | 6 |
| GO:0010411 | xyloglucan metabolic process | 6 |
| GO:0015713 | phosphoglycerate transport | 6 |
| GO:0015749 | monosaccharide transport | 6 |
| GO:0017183 | peptidyl-diphthamide biosynthetic process from peptidyl-histidine | 6 |
| GO:0019288 | isopentenyl diphosphate biosynthetic process, mevalonate-independent pathway | 6 |
| GO:0019344 | cysteine biosynthetic process | 6 |
| GO:0019432 | triglyceride biosynthetic process | 6 |
| GO:0019761 | glucosinolate biosynthetic process | 6 |
| GO:0030036 | actin cytoskeleton organization | 6 |
| GO:0030259 | lipid glycosylation | 6 |
| GO:0030834 | regulation of actin filament depolymerization | 6 |
| GO:0030968 | endoplasmic reticulum unfolded protein response | 6 |
| GO:0031119 | tRNA pseudouridine synthesis | 6 |
| GO:0032312 | regulation of ARF GTPase activity | 6 |
| GO:0034440 | lipid oxidation | 6 |
| GO:0042631 | cellular response to water deprivation | 6 |
| GO:0042732 | D-xylose metabolic process | 6 |
| GO:0042753 | positive regulation of circadian rhythm | 6 |
| GO:0044262 | cellular carbohydrate metabolic process | 6 |
| GO:0044375 | regulation of peroxisome size | 6 |
| GO:0048481 | ovule development | 6 |
| GO:0048653 | anther development | 6 |
| GO:0048766 | root hair initiation | 6 |
| GO:0051211 | anisotropic cell growth | 6 |
| GO:0052542 | defense response by callose deposition | 6 |
| GO:0055046 | microgametogenesis | 6 |
| GO:0060151 | peroxisome localization | 6 |
| GO:0071482 | cellular response to light stimulus | 6 |
| GO:0080022 | primary root development | 6 |
| GO:0080156 | mitochondrial mRNA modification | 6 |
| GO:2000039 | regulation of trichome morphogenesis | 6 |
| GO:2001141 | regulation of RNA biosynthetic process | 6 |
| GO:0000105 | histidine biosynthetic process | 5 |
| GO:0000413 | protein peptidyl-prolyl isomerization | 5 |
| GO:0000724 | double-strand break repair via homologous recombination | 5 |
| GO:0000737 | DNA catabolic process, endonucleolytic | 5 |
| GO:0005986 | sucrose biosynthetic process | 5 |
| GO:0005992 | trehalose biosynthetic process | 5 |
| GO:0006108 | malate metabolic process | 5 |
| GO:0006312 | mitotic recombination | 5 |
| GO:0006342 | chromatin silencing | 5 |
| GO:0006419 | alanyl-tRNA aminoacylation | 5 |
| GO:0006505 | GPI anchor metabolic process | 5 |
| GO:0006520 | cellular amino acid metabolic process | 5 |
| GO:0006526 | arginine biosynthetic process | 5 |
| GO:0006730 | one-carbon metabolic process | 5 |
| GO:0006788 | heme oxidation | 5 |
| GO:0006790 | sulfur compound metabolic process | 5 |
| GO:0006812 | cation transport | 5 |
| GO:0006828 | manganese ion transport | 5 |
| GO:0006885 | regulation of pH | 5 |
| GO:0007178 | transmembrane receptor protein serine/threonine kinase signaling pathway | 5 |
| GO:0007276 | gamete generation | 5 |
| GO:0009061 | anaerobic respiration | 5 |
| GO:0009097 | isoleucine biosynthetic process | 5 |
| GO:0009299 | mRNA transcription | 5 |
| GO:0009395 | phospholipid catabolic process | 5 |
| GO:0009396 | folic acid-containing compound biosynthetic process | 5 |
| GO:0009423 | chorismate biosynthetic process | 5 |
| GO:0009435 | NAD biosynthetic process | 5 |
| GO:0009704 | de-etiolation | 5 |
| GO:0009742 | brassinosteroid mediated signaling pathway | 5 |
| GO:0009755 | hormone-mediated signaling pathway | 5 |
| GO:0009821 | alkaloid biosynthetic process | 5 |
| GO:0009850 | auxin metabolic process | 5 |
| GO:0009861 | jasmonic acid and ethylene-dependent systemic resistance | 5 |
| GO:0009938 | negative regulation of gibberellic acid mediated signaling pathway | 5 |
| GO:0010052 | guard cell differentiation | 5 |
| GO:0010087 | phloem or xylem histogenesis | 5 |
| GO:0010163 | high-affinity potassium ion import | 5 |
| GO:0010218 | response to far red light | 5 |
| GO:0010233 | phloem transport | 5 |
| GO:0010236 | plastoquinone biosynthetic process | 5 |
| GO:0010252 | auxin homeostasis | 5 |
| GO:0010253 | UDP-rhamnose biosynthetic process | 5 |
| GO:0010270 | photosystem II oxygen evolving complex assembly | 5 |
| GO:0010289 | homogalacturonan biosynthetic process | 5 |
| GO:0010337 | regulation of salicylic acid metabolic process | 5 |
| GO:0010497 | plasmodesmata-mediated intercellular transport | 5 |
| GO:0010581 | regulation of starch biosynthetic process | 5 |
| GO:0010971 | positive regulation of G2/M transition of mitotic cell cycle | 5 |
| GO:0015720 | allantoin transport | 5 |
| GO:0015798 | myo-inositol transport | 5 |
| GO:0016104 | triterpenoid biosynthetic process | 5 |
| GO:0016131 | brassinosteroid metabolic process | 5 |
| GO:0016575 | histone deacetylation | 5 |
| GO:0019684 | photosynthesis, light reaction | 5 |
| GO:0019932 | second-messenger-mediated signaling | 5 |
| GO:0022619 | generative cell differentiation | 5 |
| GO:0030154 | cell differentiation | 5 |
| GO:0030301 | cholesterol transport | 5 |
| GO:0032502 | developmental process | 5 |
| GO:0033494 | ferulate metabolic process | 5 |
| GO:0034219 | carbohydrate transmembrane transport | 5 |
| GO:0034613 | cellular protein localization | 5 |
| GO:0035435 | phosphate ion transmembrane transport | 5 |
| GO:0042761 | very long-chain fatty acid biosynthetic process | 5 |
| GO:0042793 | transcription from plastid promoter | 5 |
| GO:0043181 | vacuolar sequestering | 5 |
| GO:0043484 | regulation of RNA splicing | 5 |
| GO:0043967 | histone H4 acetylation | 5 |
| GO:0044208 | 'de novo' AMP biosynthetic process | 5 |
| GO:0045493 | xylan catabolic process | 5 |
| GO:0045910 | negative regulation of DNA recombination | 5 |
| GO:0046654 | tetrahydrofolate biosynthetic process | 5 |
| GO:0046786 | viral replication complex formation and maintenance | 5 |
| GO:0048235 | pollen sperm cell differentiation | 5 |
| GO:0048437 | floral organ development | 5 |
| GO:0048442 | sepal development | 5 |
| GO:0048451 | petal formation | 5 |
| GO:0048579 | negative regulation of long-day photoperiodism, flowering | 5 |
| GO:0051013 | microtubule severing | 5 |
| GO:0051567 | histone H3-K9 methylation | 5 |
| GO:0052543 | callose deposition in cell wall | 5 |
| GO:0055071 | manganese ion homeostasis | 5 |
| GO:0071423 | malate transmembrane transport | 5 |
| GO:2000031 | regulation of salicylic acid mediated signaling pathway | 5 |
| GO:2000377 | regulation of reactive oxygen species metabolic process | 5 |
| GO:0000025 | maltose catabolic process | 4 |
| GO:0000162 | tryptophan biosynthetic process | 4 |
| GO:0000186 | activation of MAPKK activity | 4 |
| GO:0000902 | cell morphogenesis | 4 |
| GO:0000913 | preprophase band assembly | 4 |
| GO:0001676 | long-chain fatty acid metabolic process | 4 |
| GO:0006212 | uracil catabolic process | 4 |
| GO:0006269 | DNA replication, synthesis of RNA primer | 4 |
| GO:0006282 | regulation of DNA repair | 4 |
| GO:0006298 | mismatch repair | 4 |
| GO:0006420 | arginyl-tRNA aminoacylation | 4 |
| GO:0006421 | asparaginyl-tRNA aminoacylation | 4 |
| GO:0006432 | phenylalanyl-tRNA aminoacylation | 4 |
| GO:0006438 | valyl-tRNA aminoacylation | 4 |
| GO:0006479 | protein methylation | 4 |
| GO:0006536 | glutamate metabolic process | 4 |
| GO:0006782 | protoporphyrinogen IX biosynthetic process | 4 |
| GO:0006792 | regulation of sulfur utilization | 4 |
| GO:0006829 | zinc ion transport | 4 |
| GO:0006882 | cellular zinc ion homeostasis | 4 |
| GO:0007031 | peroxisome organization | 4 |
| GO:0007131 | reciprocal meiotic recombination | 4 |
| GO:0008360 | regulation of cell shape | 4 |
| GO:0009051 | pentose-phosphate shunt, oxidative branch | 4 |
| GO:0009107 | lipoate biosynthetic process | 4 |
| GO:0009113 | purine base biosynthetic process | 4 |
| GO:0009247 | glycolipid biosynthetic process | 4 |
| GO:0009554 | megasporogenesis | 4 |
| GO:0009610 | response to symbiotic fungus | 4 |
| GO:0009612 | response to mechanical stimulus | 4 |
| GO:0009699 | phenylpropanoid biosynthetic process | 4 |
| GO:0009718 | anthocyanin biosynthetic process | 4 |
| GO:0009749 | response to glucose stimulus | 4 |
| GO:0009939 | positive regulation of gibberellic acid mediated signaling pathway | 4 |
| GO:0009954 | proximal/distal pattern formation | 4 |
| GO:0009957 | epidermal cell fate specification | 4 |
| GO:0009970 | cellular response to sulfate starvation | 4 |
| GO:0010019 | chloroplast-nucleus signaling pathway | 4 |
| GO:0010021 | amylopectin biosynthetic process | 4 |
| GO:0010044 | response to aluminum ion | 4 |
| GO:0010091 | trichome branching | 4 |
| GO:0010143 | cutin biosynthetic process | 4 |
| GO:0010154 | fruit development | 4 |
| GO:0010190 | cytochrome b6f complex assembly | 4 |
| GO:0010225 | response to UV-C | 4 |
| GO:0010311 | lateral root formation | 4 |
| GO:0010312 | detoxification of zinc ion | 4 |
| GO:0010439 | regulation of glucosinolate biosynthetic process | 4 |
| GO:0010483 | pollen tube reception | 4 |
| GO:0012501 | programmed cell death | 4 |
| GO:0015670 | carbon dioxide transport | 4 |
| GO:0015688 | iron chelate transport | 4 |
| GO:0015800 | acidic amino acid transport | 4 |
| GO:0015812 | gamma-aminobutyric acid transport | 4 |
| GO:0016255 | attachment of GPI anchor to protein | 4 |
| GO:0016553 | base conversion or substitution editing | 4 |
| GO:0016571 | histone methylation | 4 |
| GO:0016973 | poly(A)+ mRNA export from nucleus | 4 |
| GO:0017148 | negative regulation of translation | 4 |
| GO:0018342 | protein prenylation | 4 |
| GO:0019953 | sexual reproduction | 4 |
| GO:0030150 | protein import into mitochondrial matrix | 4 |
| GO:0030163 | protein catabolic process | 4 |
| GO:0030433 | ER-associated protein catabolic process | 4 |
| GO:0031123 | RNA 3'-end processing | 4 |
| GO:0031204 | posttranslational protein targeting to membrane, translocation | 4 |
| GO:0031407 | oxylipin metabolic process | 4 |
| GO:0032456 | endocytic recycling | 4 |
| GO:0032499 | detection of peptidoglycan | 4 |
| GO:0032504 | multicellular organism reproduction | 4 |
| GO:0032877 | positive regulation of DNA endoreduplication | 4 |
| GO:0032973 | amino acid export | 4 |
| GO:0034605 | cellular response to heat | 4 |
| GO:0035196 | production of miRNAs involved in gene silencing by miRNA | 4 |
| GO:0042026 | protein refolding | 4 |
| GO:0042344 | indole glucosinolate catabolic process | 4 |
| GO:0042752 | regulation of circadian rhythm | 4 |
| GO:0043255 | regulation of carbohydrate biosynthetic process | 4 |
| GO:0043581 | mycelium development | 4 |
| GO:0043666 | regulation of phosphoprotein phosphatase activity | 4 |
| GO:0044036 | cell wall macromolecule metabolic process | 4 |
| GO:0045723 | positive regulation of fatty acid biosynthetic process | 4 |
| GO:0046653 | tetrahydrofolate metabolic process | 4 |
| GO:0046856 | phosphatidylinositol dephosphorylation | 4 |
| GO:0048208 | COPII vesicle coating | 4 |
| GO:0048467 | gynoecium development | 4 |
| GO:0048482 | ovule morphogenesis | 4 |
| GO:0048598 | embryonic morphogenesis | 4 |
| GO:0051028 | mRNA transport | 4 |
| GO:0051127 | positive regulation of actin nucleation | 4 |
| GO:0051205 | protein insertion into membrane | 4 |
| GO:0051262 | protein tetramerization | 4 |
| GO:0051457 | maintenance of protein location in nucleus | 4 |
| GO:0051555 | flavonol biosynthetic process | 4 |
| GO:0051865 | protein autoubiquitination | 4 |
| GO:0060918 | auxin transport | 4 |
| GO:0070084 | protein initiator methionine removal | 4 |
| GO:0070681 | glutaminyl-tRNAGln biosynthesis via transamidation | 4 |
| GO:0071577 | zinc ion transmembrane transport | 4 |
| GO:0071951 | conversion of methionyl-tRNA to N-formyl-methionyl-tRNA | 4 |
| GO:0072488 | ammonium transmembrane transport | 4 |
| GO:0090306 | spindle assembly involved in meiosis | 4 |
| GO:2000034 | regulation of seed maturation | 4 |
| GO:2000601 | positive regulation of Arp2/3 complex-mediated actin nucleation | 4 |
| GO:2000762 | regulation of phenylpropanoid metabolic process | 4 |
| GO:0000256 | allantoin catabolic process | 3 |
| GO:0000387 | spliceosomal snRNP assembly | 3 |
| GO:0000395 | nuclear mRNA 5'-splice site recognition | 3 |
| GO:0000741 | karyogamy | 3 |
| GO:0000917 | barrier septum assembly | 3 |
| GO:0001408 | guanine nucleotide transport | 3 |
| GO:0002229 | defense response to oomycetes | 3 |
| GO:0005983 | starch catabolic process | 3 |
| GO:0006006 | glucose metabolic process | 3 |
| GO:0006013 | mannose metabolic process | 3 |
| GO:0006081 | cellular aldehyde metabolic process | 3 |
| GO:0006090 | pyruvate metabolic process | 3 |
| GO:0006120 | mitochondrial electron transport, NADH to ubiquinone | 3 |
| GO:0006163 | purine nucleotide metabolic process | 3 |
| GO:0006188 | IMP biosynthetic process | 3 |
| GO:0006189 | 'de novo' IMP biosynthetic process | 3 |
| GO:0006265 | DNA topological change | 3 |
| GO:0006302 | double-strand break repair | 3 |
| GO:0006344 | maintenance of chromatin silencing | 3 |
| GO:0006370 | mRNA capping | 3 |
| GO:0006388 | tRNA splicing, via endonucleolytic cleavage and ligation | 3 |
| GO:0006417 | regulation of translation | 3 |
| GO:0006424 | glutamyl-tRNA aminoacylation | 3 |
| GO:0006428 | isoleucyl-tRNA aminoacylation | 3 |
| GO:0006461 | protein complex assembly | 3 |
| GO:0006546 | glycine catabolic process | 3 |
| GO:0006572 | tyrosine catabolic process | 3 |
| GO:0006637 | acyl-CoA metabolic process | 3 |
| GO:0006659 | phosphatidylserine biosynthetic process | 3 |
| GO:0006672 | ceramide metabolic process | 3 |
| GO:0006725 | cellular aromatic compound metabolic process | 3 |
| GO:0006750 | glutathione biosynthetic process | 3 |
| GO:0006772 | thiamine metabolic process | 3 |
| GO:0006796 | phosphate-containing compound metabolic process | 3 |
| GO:0006816 | calcium ion transport | 3 |
| GO:0006863 | purine base transport | 3 |
| GO:0006874 | cellular calcium ion homeostasis | 3 |
| GO:0006897 | endocytosis | 3 |
| GO:0006909 | phagocytosis | 3 |
| GO:0006970 | response to osmotic stress | 3 |
| GO:0006997 | nucleus organization | 3 |
| GO:0007026 | negative regulation of microtubule depolymerization | 3 |
| GO:0007029 | endoplasmic reticulum organization | 3 |
| GO:0007050 | cell cycle arrest | 3 |
| GO:0007389 | pattern specification process | 3 |
| GO:0009060 | aerobic respiration | 3 |
| GO:0009226 | nucleotide-sugar biosynthetic process | 3 |
| GO:0009234 | menaquinone biosynthetic process | 3 |
| GO:0009250 | glucan biosynthetic process | 3 |
| GO:0009308 | amine metabolic process | 3 |
| GO:0009616 | virus induced gene silencing | 3 |
| GO:0009625 | response to insect | 3 |
| GO:0009653 | anatomical structure morphogenesis | 3 |
| GO:0009662 | etioplast organization | 3 |
| GO:0009852 | auxin catabolic process | 3 |
| GO:0009866 | induced systemic resistance, ethylene mediated signaling pathway | 3 |
| GO:0009886 | post-embryonic morphogenesis | 3 |
| GO:0009926 | auxin polar transport | 3 |
| GO:0009992 | cellular water homeostasis | 3 |
| GO:0010014 | meristem initiation | 3 |
| GO:0010043 | response to zinc ion | 3 |
| GO:0010047 | fruit dehiscence | 3 |
| GO:0010072 | primary shoot apical meristem specification | 3 |
| GO:0010106 | cellular response to iron ion starvation | 3 |
| GO:0010196 | nonphotochemical quenching | 3 |
| GO:0010199 | organ boundary specification between lateral organs and the meristem | 3 |
| GO:0010204 | defense response signaling pathway, resistance gene-independent | 3 |
| GO:0010214 | seed coat development | 3 |
| GO:0010216 | maintenance of DNA methylation | 3 |
| GO:0010222 | stem vascular tissue pattern formation | 3 |
| GO:0010227 | floral organ abscission | 3 |
| GO:0010345 | suberin biosynthetic process | 3 |
| GO:0010375 | stomatal complex patterning | 3 |
| GO:0010425 | DNA methylation on cytosine within a CNG sequence | 3 |
| GO:0010508 | positive regulation of autophagy | 3 |
| GO:0010628 | positive regulation of gene expression | 3 |
| GO:0010686 | tetracyclic triterpenoid biosynthetic process | 3 |
| GO:0015671 | oxygen transport | 3 |
| GO:0015743 | malate transport | 3 |
| GO:0015819 | lysine transport | 3 |
| GO:0015884 | folic acid transport | 3 |
| GO:0016070 | RNA metabolic process | 3 |
| GO:0016075 | rRNA catabolic process | 3 |
| GO:0016485 | protein processing | 3 |
| GO:0016554 | cytidine to uridine editing | 3 |
| GO:0016579 | protein deubiquitination | 3 |
| GO:0018023 | peptidyl-lysine trimethylation | 3 |
| GO:0018149 | peptide cross-linking | 3 |
| GO:0018298 | protein-chromophore linkage | 3 |
| GO:0019064 | viral envelope fusion with host membrane | 3 |
| GO:0019079 | viral genome replication | 3 |
| GO:0019354 | siroheme biosynthetic process | 3 |
| GO:0019408 | dolichol biosynthetic process | 3 |
| GO:0019538 | protein metabolic process | 3 |
| GO:0019745 | pentacyclic triterpenoid biosynthetic process | 3 |
| GO:0019853 | L-ascorbic acid biosynthetic process | 3 |
| GO:0022904 | respiratory electron transport chain | 3 |
| GO:0030422 | production of siRNA involved in RNA interference | 3 |
| GO:0030833 | regulation of actin filament polymerization | 3 |
| GO:0032268 | regulation of cellular protein metabolic process | 3 |
| GO:0032319 | regulation of Rho GTPase activity | 3 |
| GO:0033306 | phytol metabolic process | 3 |
| GO:0034059 | response to anoxia | 3 |
| GO:0034196 | acylglycerol transport | 3 |
| GO:0034434 | sterol esterification | 3 |
| GO:0034508 | centromere complex assembly | 3 |
| GO:0034599 | cellular response to oxidative stress | 3 |
| GO:0035672 | oligopeptide transmembrane transport | 3 |
| GO:0035725 | sodium ion transmembrane transport | 3 |
| GO:0042023 | DNA endoreduplication | 3 |
| GO:0042176 | regulation of protein catabolic process | 3 |
| GO:0042335 | cuticle development | 3 |
| GO:0042372 | phylloquinone biosynthetic process | 3 |
| GO:0042542 | response to hydrogen peroxide | 3 |
| GO:0042823 | pyridoxal phosphate biosynthetic process | 3 |
| GO:0043068 | positive regulation of programmed cell death | 3 |
| GO:0043085 | positive regulation of catalytic activity | 3 |
| GO:0043090 | amino acid import | 3 |
| GO:0043388 | positive regulation of DNA binding | 3 |
| GO:0043401 | steroid hormone mediated signaling pathway | 3 |
| GO:0043447 | alkane biosynthetic process | 3 |
| GO:0043462 | regulation of ATPase activity | 3 |
| GO:0043631 | RNA polyadenylation | 3 |
| GO:0045038 | protein import into chloroplast thylakoid membrane | 3 |
| GO:0045087 | innate immune response | 3 |
| GO:0045185 | maintenance of protein location | 3 |
| GO:0045333 | cellular respiration | 3 |
| GO:0045739 | positive regulation of DNA repair | 3 |
| GO:0046183 | L-idonate catabolic process | 3 |
| GO:0046215 | siderophore catabolic process | 3 |
| GO:0046274 | lignin catabolic process | 3 |
| GO:0046836 | glycolipid transport | 3 |
| GO:0048255 | mRNA stabilization | 3 |
| GO:0048359 | mucilage metabolic process involved seed coat development | 3 |
| GO:0048446 | petal morphogenesis | 3 |
| GO:0048453 | sepal formation | 3 |
| GO:0048507 | meristem development | 3 |
| GO:0048527 | lateral root development | 3 |
| GO:0048564 | photosystem I assembly | 3 |
| GO:0050776 | regulation of immune response | 3 |
| GO:0051016 | barbed-end actin filament capping | 3 |
| GO:0051085 | chaperone mediated protein folding requiring cofactor | 3 |
| GO:0051297 | centrosome organization | 3 |
| GO:0051604 | protein maturation | 3 |
| GO:0051726 | regulation of cell cycle | 3 |
| GO:0055063 | sulfate ion homeostasis | 3 |
| GO:0055069 | zinc ion homeostasis | 3 |
| GO:0060548 | negative regulation of cell death | 3 |
| GO:0070328 | triglyceride homeostasis | 3 |
| GO:0070933 | histone H4 deacetylation | 3 |
| GO:0071215 | cellular response to abscisic acid stimulus | 3 |
| GO:0071266 | 'de novo' L-methionine biosynthetic process | 3 |
| GO:0071311 | cellular response to acetate | 3 |
| GO:0071365 | cellular response to auxin stimulus | 3 |
| GO:0071366 | cellular response to indolebutyric acid stimulus | 3 |
| GO:0071483 | cellular response to blue light | 3 |
| GO:0071786 | endoplasmic reticulum tubular network organization | 3 |
| GO:0080009 | mRNA methylation | 3 |
| GO:0080111 | DNA demethylation | 3 |
| GO:0080141 | regulation of jasmonic acid biosynthetic process | 3 |
| GO:0080153 | negative regulation of reductive pentose-phosphate cycle | 3 |
| GO:0090309 | positive regulation of methylation-dependent chromatin silencing | 3 |
| GO:0090333 | regulation of stomatal closure | 3 |
| GO:0090408 | phloem nitrate loading | 3 |
| GO:2000014 | regulation of endosperm development | 3 |
| GO:2000067 | regulation of root morphogenesis | 3 |
| GO:0000023 | maltose metabolic process | 2 |
| GO:0000024 | maltose biosynthetic process | 2 |
| GO:0000042 | protein targeting to Golgi | 2 |
| GO:0000076 | DNA replication checkpoint | 2 |
| GO:0000077 | DNA damage checkpoint | 2 |
| GO:0000271 | polysaccharide biosynthetic process | 2 |
| GO:0000280 | nuclear division | 2 |
| GO:0000461 | endonucleolytic cleavage to generate mature 3'-end of SSU-rRNA from (SSU-rRNA, 5.8S rRNA, LSU-rRNA) | 2 |
| GO:0000462 | maturation of SSU-rRNA from tricistronic rRNA transcript (SSU-rRNA, 5.8S rRNA, LSU-rRNA) | 2 |
| GO:0000489 | maturation of SSU-rRNA from tetracistronic rRNA transcript (SSU-rRNA, LSU-rRNA, 4.5S-rRNA, 5S-rRNA) | 2 |
| GO:0000919 | cell plate assembly | 2 |
| GO:0001708 | cell fate specification | 2 |
| GO:0001887 | selenium compound metabolic process | 2 |
| GO:0002053 | positive regulation of mesenchymal cell proliferation | 2 |
| GO:0002100 | tRNA wobble adenosine to inosine editing | 2 |
| GO:0002213 | defense response to insect | 2 |
| GO:0005985 | sucrose metabolic process | 2 |
| GO:0006004 | fucose metabolic process | 2 |
| GO:0006032 | chitin catabolic process | 2 |
| GO:0006047 | UDP-N-acetylglucosamine metabolic process | 2 |
| GO:0006069 | ethanol oxidation | 2 |
| GO:0006075 | (1->3)-beta-D-glucan biosynthetic process | 2 |
| GO:0006109 | regulation of carbohydrate metabolic process | 2 |
| GO:0006144 | purine base metabolic process | 2 |
| GO:0006148 | inosine catabolic process | 2 |
| GO:0006222 | UMP biosynthetic process | 2 |
| GO:0006241 | CTP biosynthetic process | 2 |
| GO:0006259 | DNA metabolic process | 2 |
| GO:0006266 | DNA ligation | 2 |
| GO:0006275 | regulation of DNA replication | 2 |
| GO:0006383 | transcription from RNA polymerase III promoter | 2 |
| GO:0006406 | mRNA export from nucleus | 2 |
| GO:0006418 | tRNA aminoacylation for protein translation | 2 |
| GO:0006423 | cysteinyl-tRNA aminoacylation | 2 |
| GO:0006446 | regulation of translational initiation | 2 |
| GO:0006476 | protein deacetylation | 2 |
| GO:0006481 | C-terminal protein methylation | 2 |
| GO:0006535 | cysteine biosynthetic process from serine | 2 |
| GO:0006551 | leucine metabolic process | 2 |
| GO:0006580 | ethanolamine metabolic process | 2 |
| GO:0006596 | polyamine biosynthetic process | 2 |
| GO:0006621 | protein retention in ER lumen | 2 |
| GO:0006646 | phosphatidylethanolamine biosynthetic process | 2 |
| GO:0006651 | diacylglycerol biosynthetic process | 2 |
| GO:0006665 | sphingolipid metabolic process | 2 |
| GO:0006754 | ATP biosynthetic process | 2 |
| GO:0006760 | folic acid-containing compound metabolic process | 2 |
| GO:0006805 | xenobiotic metabolic process | 2 |
| GO:0006817 | phosphate ion transport | 2 |
| GO:0006879 | cellular iron ion homeostasis | 2 |
| GO:0006890 | retrograde vesicle-mediated transport, Golgi to ER | 2 |
| GO:0006896 | Golgi to vacuole transport | 2 |
| GO:0006898 | receptor-mediated endocytosis | 2 |
| GO:0006974 | response to DNA damage stimulus | 2 |
| GO:0007005 | mitochondrion organization | 2 |
| GO:0007021 | tubulin complex assembly | 2 |
| GO:0007032 | endosome organization | 2 |
| GO:0007094 | mitotic cell cycle spindle assembly checkpoint | 2 |
| GO:0007141 | male meiosis I | 2 |
| GO:0007186 | G-protein coupled receptor signaling pathway | 2 |
| GO:0007283 | spermatogenesis | 2 |
| GO:0008033 | tRNA processing | 2 |
| GO:0008154 | actin polymerization or depolymerization | 2 |
| GO:0008361 | regulation of cell size | 2 |
| GO:0008535 | respiratory chain complex IV assembly | 2 |
| GO:0008616 | queuosine biosynthetic process | 2 |
| GO:0009102 | biotin biosynthetic process | 2 |
| GO:0009156 | ribonucleoside monophosphate biosynthetic process | 2 |
| GO:0009157 | deoxyribonucleoside monophosphate biosynthetic process | 2 |
| GO:0009244 | lipopolysaccharide core region biosynthetic process | 2 |
| GO:0009267 | cellular response to starvation | 2 |
| GO:0009298 | GDP-mannose biosynthetic process | 2 |
| GO:0009303 | rRNA transcription | 2 |
| GO:0009311 | oligosaccharide metabolic process | 2 |
| GO:0009405 | pathogenesis | 2 |
| GO:0009583 | detection of light stimulus | 2 |
| GO:0009585 | red, far-red light phototransduction | 2 |
| GO:0009645 | response to low light intensity stimulus | 2 |
| GO:0009652 | thigmotropism | 2 |
| GO:0009688 | abscisic acid biosynthetic process | 2 |
| GO:0009691 | cytokinin biosynthetic process | 2 |
| GO:0009722 | detection of cytokinin stimulus | 2 |
| GO:0009740 | gibberellic acid mediated signaling pathway | 2 |
| GO:0009768 | photosynthesis, light harvesting in photosystem I | 2 |
| GO:0009788 | negative regulation of abscisic acid mediated signaling pathway | 2 |
| GO:0009792 | embryo development ending in birth or egg hatching | 2 |
| GO:0009801 | cinnamic acid ester metabolic process | 2 |
| GO:0009820 | alkaloid metabolic process | 2 |
| GO:0009828 | plant-type cell wall loosening | 2 |
| GO:0009904 | chloroplast accumulation movement | 2 |
| GO:0009942 | longitudinal axis specification | 2 |
| GO:0009969 | xyloglucan biosynthetic process | 2 |
| GO:0010015 | root morphogenesis | 2 |
| GO:0010028 | xanthophyll cycle | 2 |
| GO:0010031 | circumnutation | 2 |
| GO:0010037 | response to carbon dioxide | 2 |
| GO:0010051 | xylem and phloem pattern formation | 2 |
| GO:0010081 | regulation of inflorescence meristem growth | 2 |
| GO:0010082 | regulation of root meristem growth | 2 |
| GO:0010093 | specification of floral organ identity | 2 |
| GO:0010099 | regulation of photomorphogenesis | 2 |
| GO:0010100 | negative regulation of photomorphogenesis | 2 |
| GO:0010111 | glyoxysome organization | 2 |
| GO:0010184 | cytokinin transport | 2 |
| GO:0010205 | photoinhibition | 2 |
| GO:0010223 | secondary shoot formation | 2 |
| GO:0010260 | organ senescence | 2 |
| GO:0010268 | brassinosteroid homeostasis | 2 |
| GO:0010271 | regulation of chlorophyll catabolic process | 2 |
| GO:0010306 | rhamnogalacturonan II biosynthetic process | 2 |
| GO:0010343 | singlet oxygen-mediated programmed cell death | 2 |
| GO:0010352 | lithium ion export | 2 |
| GO:0010366 | negative regulation of ethylene biosynthetic process | 2 |
| GO:0010387 | signalosome assembly | 2 |
| GO:0010388 | cullin deneddylation | 2 |
| GO:0010394 | homogalacturonan metabolic process | 2 |
| GO:0010423 | negative regulation of brassinosteroid biosynthetic process | 2 |
| GO:0010493 | Lewis a epitope biosynthetic process | 2 |
| GO:0010498 | proteasomal protein catabolic process | 2 |
| GO:0010582 | floral meristem determinacy | 2 |
| GO:0010588 | cotyledon vascular tissue pattern formation | 2 |
| GO:0010599 | production of lsiRNA involved in RNA interference | 2 |
| GO:0010951 | negative regulation of endopeptidase activity | 2 |
| GO:0010964 | regulation of chromatin silencing by small RNA | 2 |
| GO:0015689 | molybdate ion transport | 2 |
| GO:0015739 | sialic acid transport | 2 |
| GO:0015780 | nucleotide-sugar transport | 2 |
| GO:0015784 | GDP-mannose transport | 2 |
| GO:0015846 | polyamine transport | 2 |
| GO:0015916 | fatty-acyl-CoA transport | 2 |
| GO:0015931 | nucleobase-containing compound transport | 2 |
| GO:0015940 | pantothenate biosynthetic process | 2 |
| GO:0015967 | diadenosine tetraphosphate catabolic process | 2 |
| GO:0015977 | carbon fixation | 2 |
| GO:0016050 | vesicle organization | 2 |
| GO:0016121 | carotene catabolic process | 2 |
| GO:0016123 | xanthophyll biosynthetic process | 2 |
| GO:0016444 | somatic cell DNA recombination | 2 |
| GO:0016572 | histone phosphorylation | 2 |
| GO:0017004 | cytochrome complex assembly | 2 |
| GO:0017145 | stem cell division | 2 |
| GO:0018105 | peptidyl-serine phosphorylation | 2 |
| GO:0018258 | protein O-linked glycosylation via hydroxyproline | 2 |
| GO:0019310 | inositol catabolic process | 2 |
| GO:0019365 | pyridine nucleotide salvage | 2 |
| GO:0019427 | acetyl-CoA biosynthetic process from acetate | 2 |
| GO:0019478 | D-amino acid catabolic process | 2 |
| GO:0019541 | propionate metabolic process | 2 |
| GO:0019676 | ammonia assimilation cycle | 2 |
| GO:0019752 | carboxylic acid metabolic process | 2 |
| GO:0019760 | glucosinolate metabolic process | 2 |
| GO:0019805 | quinolinate biosynthetic process | 2 |
| GO:0022603 | regulation of anatomical structure morphogenesis | 2 |
| GO:0030007 | cellular potassium ion homeostasis | 2 |
| GO:0030245 | cellulose catabolic process | 2 |
| GO:0030308 | negative regulation of cell growth | 2 |
| GO:0030336 | negative regulation of cell migration | 2 |
| GO:0030388 | fructose 1,6-bisphosphate metabolic process | 2 |
| GO:0030497 | fatty acid elongation | 2 |
| GO:0031023 | microtubule organizing center organization | 2 |
| GO:0031539 | positive regulation of anthocyanin metabolic process | 2 |
| GO:0031930 | mitochondria-nucleus signaling pathway | 2 |
| GO:0032260 | response to jasmonic acid stimulus involved in jasmonic acid and ethylene-dependent systemic resistance | 2 |
| GO:0032509 | endosome transport via multivesicular body sorting pathway | 2 |
| GO:0032781 | positive regulation of ATPase activity | 2 |
| GO:0033169 | histone H3-K9 demethylation | 2 |
| GO:0033388 | putrescine biosynthetic process from arginine | 2 |
| GO:0033528 | S-methylmethionine cycle | 2 |
| GO:0034427 | nuclear-transcribed mRNA catabolic process, exonucleolytic, 3'-5' | 2 |
| GO:0034755 | iron ion transmembrane transport | 2 |
| GO:0034765 | regulation of ion transmembrane transport | 2 |
| GO:0035246 | peptidyl-arginine N-methylation | 2 |
| GO:0035265 | organ growth | 2 |
| GO:0035280 | miRNA loading onto RISC involved in gene silencing by miRNA | 2 |
| GO:0035445 | borate transmembrane transport | 2 |
| GO:0042052 | rhabdomere development | 2 |
| GO:0042147 | retrograde transport, endosome to Golgi | 2 |
| GO:0042276 | error-prone translesion synthesis | 2 |
| GO:0042318 | penicillin biosynthetic process | 2 |
| GO:0042353 | fucose biosynthetic process | 2 |
| GO:0042547 | cell wall modification involved in multidimensional cell growth | 2 |
| GO:0042594 | response to starvation | 2 |
| GO:0042759 | long-chain fatty acid biosynthetic process | 2 |
| GO:0043043 | peptide biosynthetic process | 2 |
| GO:0043103 | hypoxanthine salvage | 2 |
| GO:0043461 | proton-transporting ATP synthase complex assembly | 2 |
| GO:0043562 | cellular response to nitrogen levels | 2 |
| GO:0043574 | peroxisomal transport | 2 |
| GO:0043985 | histone H4-R3 methylation | 2 |
| GO:0044205 | 'de novo' UMP biosynthetic process | 2 |
| GO:0045010 | actin nucleation | 2 |
| GO:0045036 | protein targeting to chloroplast | 2 |
| GO:0045040 | protein import into mitochondrial outer membrane | 2 |
| GO:0045324 | late endosome to vacuole transport | 2 |
| GO:0045596 | negative regulation of cell differentiation | 2 |
| GO:0045980 | negative regulation of nucleotide metabolic process | 2 |
| GO:0046373 | L-arabinose metabolic process | 2 |
| GO:0046488 | phosphatidylinositol metabolic process | 2 |
| GO:0046500 | S-adenosylmethionine metabolic process | 2 |
| GO:0046739 | spread of virus in host | 2 |
| GO:0048280 | vesicle fusion with Golgi apparatus | 2 |
| GO:0048363 | mucilage pectin metabolic process | 2 |
| GO:0048513 | organ development | 2 |
| GO:0048519 | negative regulation of biological process | 2 |
| GO:0048589 | developmental growth | 2 |
| GO:0048629 | trichome patterning | 2 |
| GO:0048700 | acquisition of desiccation tolerance | 2 |
| GO:0050896 | response to stimulus | 2 |
| GO:0050982 | detection of mechanical stimulus | 2 |
| GO:0051026 | chiasma assembly | 2 |
| GO:0051131 | chaperone-mediated protein complex assembly | 2 |
| GO:0051260 | protein homooligomerization | 2 |
| GO:0051603 | proteolysis involved in cellular protein catabolic process | 2 |
| GO:0051650 | establishment of vesicle localization | 2 |
| GO:0051776 | detection of redox state | 2 |
| GO:0051928 | positive regulation of calcium ion transport | 2 |
| GO:0052546 | cell wall pectin metabolic process | 2 |
| GO:0055062 | phosphate ion homeostasis | 2 |
| GO:0060316 | positive regulation of ryanodine-sensitive calcium-release channel activity | 2 |
| GO:0061087 | positive regulation of histone H3-K27 methylation | 2 |
| GO:0070207 | protein homotrimerization | 2 |
| GO:0070483 | detection of hypoxia | 2 |
| GO:0070509 | calcium ion import | 2 |
| GO:0070838 | divalent metal ion transport | 2 |
| GO:0070981 | L-asparagine biosynthetic process | 2 |
| GO:0071369 | cellular response to ethylene stimulus | 2 |
| GO:0071398 | cellular response to fatty acid | 2 |
| GO:0071486 | cellular response to high light intensity | 2 |
| GO:0071555 | cell wall organization | 2 |
| GO:0071732 | cellular response to nitric oxide | 2 |
| GO:0072334 | UDP-galactose transmembrane transport | 2 |
| GO:0072593 | reactive oxygen species metabolic process | 2 |
| GO:0080092 | regulation of pollen tube growth | 2 |
| GO:2000032 | regulation of secondary shoot formation | 2 |
| GO:0000002 | mitochondrial genome maintenance | 1 |
| GO:0000045 | autophagic vacuole assembly | 1 |
| GO:0000059 | protein import into nucleus, docking | 1 |
| GO:0000087 | M phase of mitotic cell cycle | 1 |
| GO:0000122 | negative regulation of transcription from RNA polymerase II promoter | 1 |
| GO:0000128 | flocculation | 1 |
| GO:0000209 | protein polyubiquitination | 1 |
| GO:0000244 | assembly of spliceosomal tri-snRNP | 1 |
| GO:0000272 | polysaccharide catabolic process | 1 |
| GO:0000302 | response to reactive oxygen species | 1 |
| GO:0000447 | endonucleolytic cleavage in ITS1 to separate SSU-rRNA from 5.8S rRNA and LSU-rRNA from tricistronic rRNA transcript (SSU-rRNA, 5.8S rRNA, LSU-rRNA) | 1 |
| GO:0000463 | maturation of LSU-rRNA from tricistronic rRNA transcript (SSU-rRNA, 5.8S rRNA, LSU-rRNA) | 1 |
| GO:0000491 | small nucleolar ribonucleoprotein complex assembly | 1 |
| GO:0000712 | resolution of meiotic recombination intermediates | 1 |
| GO:0000735 | removal of nonhomologous ends | 1 |
| GO:0000914 | phragmoplast assembly | 1 |
| GO:0000963 | mitochondrial RNA processing | 1 |
| GO:0001189 | RNA polymerase I transcriptional preinitiation complex assembly at the promoter for the nuclear large rRNA transcript | 1 |
| GO:0001525 | angiogenesis | 1 |
| GO:0001702 | gastrulation with mouth forming second | 1 |
| GO:0001964 | startle response | 1 |
| GO:0002077 | acrosome matrix dispersal | 1 |
| GO:0002230 | positive regulation of defense response to virus by host | 1 |
| GO:0003002 | regionalization | 1 |
| GO:0003006 | developmental process involved in reproduction | 1 |
| GO:0005980 | glycogen catabolic process | 1 |
| GO:0005993 | trehalose catabolic process | 1 |
| GO:0006003 | fructose 2,6-bisphosphate metabolic process | 1 |
| GO:0006030 | chitin metabolic process | 1 |
| GO:0006031 | chitin biosynthetic process | 1 |
| GO:0006048 | UDP-N-acetylglucosamine biosynthetic process | 1 |
| GO:0006072 | glycerol-3-phosphate metabolic process | 1 |
| GO:0006097 | glyoxylate cycle | 1 |
| GO:0006101 | citrate metabolic process | 1 |
| GO:0006114 | glycerol biosynthetic process | 1 |
| GO:0006166 | purine ribonucleoside salvage | 1 |
| GO:0006233 | dTDP biosynthetic process | 1 |
| GO:0006267 | pre-replicative complex assembly | 1 |
| GO:0006283 | transcription-coupled nucleotide-excision repair | 1 |
| GO:0006290 | pyrimidine dimer repair | 1 |
| GO:0006338 | chromatin remodeling | 1 |
| GO:0006349 | regulation of gene expression by genetic imprinting | 1 |
| GO:0006366 | transcription from RNA polymerase II promoter | 1 |
| GO:0006379 | mRNA cleavage | 1 |
| GO:0006399 | tRNA metabolic process | 1 |
| GO:0006401 | RNA catabolic process | 1 |
| GO:0006422 | aspartyl-tRNA aminoacylation | 1 |
| GO:0006426 | glycyl-tRNA aminoacylation | 1 |
| GO:0006430 | lysyl-tRNA aminoacylation | 1 |
| GO:0006434 | seryl-tRNA aminoacylation | 1 |
| GO:0006435 | threonyl-tRNA aminoacylation | 1 |
| GO:0006436 | tryptophanyl-tRNA aminoacylation | 1 |
| GO:0006437 | tyrosyl-tRNA aminoacylation | 1 |
| GO:0006471 | protein ADP-ribosylation | 1 |
| GO:0006490 | oligosaccharide-lipid intermediate biosynthetic process | 1 |
| GO:0006493 | protein O-linked glycosylation | 1 |
| GO:0006534 | cysteine metabolic process | 1 |
| GO:0006537 | glutamate biosynthetic process | 1 |
| GO:0006559 | L-phenylalanine catabolic process | 1 |
| GO:0006563 | L-serine metabolic process | 1 |
| GO:0006568 | tryptophan metabolic process | 1 |
| GO:0006574 | valine catabolic process | 1 |
| GO:0006591 | ornithine metabolic process | 1 |
| GO:0006624 | vacuolar protein processing | 1 |
| GO:0006625 | protein targeting to peroxisome | 1 |
| GO:0006636 | unsaturated fatty acid biosynthetic process | 1 |
| GO:0006650 | glycerophospholipid metabolic process | 1 |
| GO:0006657 | CDP-choline pathway | 1 |
| GO:0006723 | cuticle hydrocarbon biosynthetic process | 1 |
| GO:0006729 | tetrahydrobiopterin biosynthetic process | 1 |
| GO:0006739 | NADP metabolic process | 1 |
| GO:0006741 | NADP biosynthetic process | 1 |
| GO:0006751 | glutathione catabolic process | 1 |
| GO:0006761 | dihydrofolate biosynthetic process | 1 |
| GO:0006779 | porphyrin-containing compound biosynthetic process | 1 |
| GO:0006783 | heme biosynthetic process | 1 |
| GO:0006784 | heme a biosynthetic process | 1 |
| GO:0006820 | anion transport | 1 |
| GO:0006824 | cobalt ion transport | 1 |
| GO:0006826 | iron ion transport | 1 |
| GO:0006835 | dicarboxylic acid transport | 1 |
| GO:0006839 | mitochondrial transport | 1 |
| GO:0006873 | cellular ion homeostasis | 1 |
| GO:0006949 | syncytium formation | 1 |
| GO:0006954 | inflammatory response | 1 |
| GO:0007033 | vacuole organization | 1 |
| GO:0007040 | lysosome organization | 1 |
| GO:0007052 | mitotic spindle organization | 1 |
| GO:0007059 | chromosome segregation | 1 |
| GO:0007065 | male meiosis sister chromatid cohesion | 1 |
| GO:0007066 | female meiosis sister chromatid cohesion | 1 |
| GO:0007108 | cytokinesis, initiation of separation | 1 |
| GO:0007112 | male meiosis cytokinesis | 1 |
| GO:0007126 | meiosis | 1 |
| GO:0007135 | meiosis II | 1 |
| GO:0007190 | activation of adenylate cyclase activity | 1 |
| GO:0007218 | neuropeptide signaling pathway | 1 |
| GO:0007399 | nervous system development | 1 |
| GO:0008285 | negative regulation of cell proliferation | 1 |
| GO:0008340 | determination of adult lifespan | 1 |
| GO:0008612 | peptidyl-lysine modification to hypusine | 1 |
| GO:0008615 | pyridoxine biosynthetic process | 1 |
| GO:0009052 | pentose-phosphate shunt, non-oxidative branch | 1 |
| GO:0009067 | aspartate family amino acid biosynthetic process | 1 |
| GO:0009073 | aromatic amino acid family biosynthetic process | 1 |
| GO:0009081 | branched chain family amino acid metabolic process | 1 |
| GO:0009094 | L-phenylalanine biosynthetic process | 1 |
| GO:0009098 | leucine biosynthetic process | 1 |
| GO:0009103 | lipopolysaccharide biosynthetic process | 1 |
| GO:0009108 | coenzyme biosynthetic process | 1 |
| GO:0009124 | nucleoside monophosphate biosynthetic process | 1 |
| GO:0009204 | deoxyribonucleoside triphosphate catabolic process | 1 |
| GO:0009228 | thiamine biosynthetic process | 1 |
| GO:0009252 | peptidoglycan biosynthetic process | 1 |
| GO:0009268 | response to pH | 1 |
| GO:0009312 | oligosaccharide biosynthetic process | 1 |
| GO:0009314 | response to radiation | 1 |
| GO:0009415 | response to water | 1 |
| GO:0009440 | cyanate catabolic process | 1 |
| GO:0009446 | putrescine biosynthetic process | 1 |
| GO:0009450 | gamma-aminobutyric acid catabolic process | 1 |
| GO:0009556 | microsporogenesis | 1 |
| GO:0009608 | response to symbiont | 1 |
| GO:0009636 | response to toxin | 1 |
| GO:0009643 | photosynthetic acclimation | 1 |
| GO:0009647 | skotomorphogenesis | 1 |
| GO:0009657 | plastid organization | 1 |
| GO:0009686 | gibberellin biosynthetic process | 1 |
| GO:0009694 | jasmonic acid metabolic process | 1 |
| GO:0009759 | indole glucosinolate biosynthetic process | 1 |
| GO:0009772 | photosynthetic electron transport in photosystem II | 1 |
| GO:0009800 | cinnamic acid biosynthetic process | 1 |
| GO:0009812 | flavonoid metabolic process | 1 |
| GO:0009819 | drought recovery | 1 |
| GO:0009823 | cytokinin catabolic process | 1 |
| GO:0009831 | plant-type cell wall modification involved in multidimensional cell growth | 1 |
| GO:0009854 | oxidative photosynthetic carbon pathway | 1 |
| GO:0009877 | nodulation | 1 |
| GO:0009887 | organ morphogenesis | 1 |
| GO:0009893 | positive regulation of metabolic process | 1 |
| GO:0009920 | cell plate formation involved in plant-type cell wall biogenesis | 1 |
| GO:0009933 | meristem structural organization | 1 |
| GO:0009945 | radial axis specification | 1 |
| GO:0009956 | radial pattern formation | 1 |
| GO:0009959 | negative gravitropism | 1 |
| GO:0009966 | regulation of signal transduction | 1 |
| GO:0009967 | positive regulation of signal transduction | 1 |
| GO:0009972 | cytidine deamination | 1 |
| GO:0010017 | red or far-red light signaling pathway | 1 |
| GO:0010023 | proanthocyanidin biosynthetic process | 1 |
| GO:0010067 | procambium histogenesis | 1 |
| GO:0010073 | meristem maintenance | 1 |
| GO:0010076 | maintenance of floral meristem identity | 1 |
| GO:0010088 | phloem development | 1 |
| GO:0010098 | suspensor development | 1 |
| GO:0010105 | negative regulation of ethylene mediated signaling pathway | 1 |
| GO:0010117 | photoprotection | 1 |
| GO:0010159 | specification of organ position | 1 |
| GO:0010160 | formation of organ boundary | 1 |
| GO:0010167 | response to nitrate | 1 |
| GO:0010226 | response to lithium ion | 1 |
| GO:0010229 | inflorescence development | 1 |
| GO:0010248 | establishment or maintenance of transmembrane electrochemical gradient | 1 |
| GO:0010258 | NADH dehydrogenase complex (plastoquinone) assembly | 1 |
| GO:0010269 | response to selenium ion | 1 |
| GO:0010288 | response to lead ion | 1 |
| GO:0010321 | regulation of vegetative phase change | 1 |
| GO:0010324 | membrane invagination | 1 |
| GO:0010351 | lithium ion transport | 1 |
| GO:0010358 | leaf shaping | 1 |
| GO:0010371 | regulation of gibberellin biosynthetic process | 1 |
| GO:0010389 | regulation of G2/M transition of mitotic cell cycle | 1 |
| GO:0010390 | histone monoubiquitination | 1 |
| GO:0010422 | regulation of brassinosteroid biosynthetic process | 1 |
| GO:0010449 | root meristem growth | 1 |
| GO:0010478 | chlororespiration | 1 |
| GO:0010492 | maintenance of shoot apical meristem identity | 1 |
| GO:0010500 | transmitting tissue development | 1 |
| GO:0010501 | RNA secondary structure unwinding | 1 |
| GO:0010541 | acropetal auxin transport | 1 |
| GO:0010583 | response to cyclopentenone | 1 |
| GO:0010589 | leaf proximal/distal pattern formation | 1 |
| GO:0010928 | regulation of auxin mediated signaling pathway | 1 |
| GO:0015684 | ferrous iron transport | 1 |
| GO:0015692 | lead ion transport | 1 |
| GO:0015707 | nitrite transport | 1 |
| GO:0015748 | organophosphate ester transport | 1 |
| GO:0015757 | galactose transport | 1 |
| GO:0015764 | N-acetylglucosamine transport | 1 |
| GO:0015783 | GDP-fucose transport | 1 |
| GO:0015791 | polyol transport | 1 |
| GO:0015801 | aromatic amino acid transport | 1 |
| GO:0015810 | aspartate transport | 1 |
| GO:0015817 | histidine transport | 1 |
| GO:0015851 | nucleobase transport | 1 |
| GO:0015854 | guanine transport | 1 |
| GO:0015867 | ATP transport | 1 |
| GO:0015914 | phospholipid transport | 1 |
| GO:0015969 | guanosine tetraphosphate metabolic process | 1 |
| GO:0016024 | CDP-diacylglycerol biosynthetic process | 1 |
| GO:0016052 | carbohydrate catabolic process | 1 |
| GO:0016116 | carotenoid metabolic process | 1 |
| GO:0016120 | carotene biosynthetic process | 1 |
| GO:0016125 | sterol metabolic process | 1 |
| GO:0016126 | sterol biosynthetic process | 1 |
| GO:0016180 | snRNA processing | 1 |
| GO:0016233 | telomere capping | 1 |
| GO:0016458 | gene silencing | 1 |
| GO:0016477 | cell migration | 1 |
| GO:0016480 | negative regulation of transcription from RNA polymerase III promoter | 1 |
| GO:0016539 | intein-mediated protein splicing | 1 |
| GO:0016560 | protein import into peroxisome matrix, docking | 1 |
| GO:0016570 | histone modification | 1 |
| GO:0016578 | histone deubiquitination | 1 |
| GO:0018192 | enzyme active site formation via L-cysteine persulfide | 1 |
| GO:0018272 | protein-pyridoxal-5-phosphate linkage via peptidyl-N6-pyridoxal phosphate-L-lysine | 1 |
| GO:0019249 | lactate biosynthetic process | 1 |
| GO:0019290 | siderophore biosynthetic process | 1 |
| GO:0019295 | coenzyme M biosynthetic process | 1 |
| GO:0019307 | mannose biosynthetic process | 1 |
| GO:0019318 | hexose metabolic process | 1 |
| GO:0019358 | nicotinate nucleotide salvage | 1 |
| GO:0019370 | leukotriene biosynthetic process | 1 |
| GO:0019388 | galactose catabolic process | 1 |
| GO:0019424 | sulfide oxidation, using siroheme sulfite reductase | 1 |
| GO:0019439 | aromatic compound catabolic process | 1 |
| GO:0019464 | glycine decarboxylation via glycine cleavage system | 1 |
| GO:0019605 | butyrate metabolic process | 1 |
| GO:0019629 | propionate catabolic process, 2-methylcitrate cycle | 1 |
| GO:0019673 | GDP-mannose metabolic process | 1 |
| GO:0019674 | NAD metabolic process | 1 |
| GO:0019835 | cytolysis | 1 |
| GO:0023014 | signal transduction via phosphorylation event | 1 |
| GO:0030003 | cellular cation homeostasis | 1 |
| GO:0030097 | hemopoiesis | 1 |
| GO:0030148 | sphingolipid biosynthetic process | 1 |
| GO:0030261 | chromosome condensation | 1 |
| GO:0030974 | thiamine pyrophosphate transport | 1 |
| GO:0031053 | primary miRNA processing | 1 |
| GO:0031214 | biomineral tissue development | 1 |
| GO:0031222 | arabinan catabolic process | 1 |
| GO:0031505 | fungal-type cell wall organization | 1 |
| GO:0031640 | killing of cells of other organism | 1 |
| GO:0032264 | IMP salvage | 1 |
| GO:0032321 | positive regulation of Rho GTPase activity | 1 |
| GO:0032365 | intracellular lipid transport | 1 |
| GO:0032418 | lysosome localization | 1 |
| GO:0032774 | RNA biosynthetic process | 1 |
| GO:0032875 | regulation of DNA endoreduplication | 1 |
| GO:0032922 | circadian regulation of gene expression | 1 |
| GO:0032955 | regulation of barrier septum assembly | 1 |
| GO:0033014 | tetrapyrrole biosynthetic process | 1 |
| GO:0033303 | quercetin O-glucoside biosynthetic process | 1 |
| GO:0033345 | asparagine catabolic process via L-aspartate | 1 |
| GO:0033354 | chlorophyll cycle | 1 |
| GO:0033356 | UDP-L-arabinose metabolic process | 1 |
| GO:0033358 | UDP-L-arabinose biosynthetic process | 1 |
| GO:0033466 | trans-zeatin biosynthetic process | 1 |
| GO:0033468 | CMP-keto-3-deoxy-D-manno-octulosonic acid biosynthetic process | 1 |
| GO:0033473 | indoleacetic acid conjugate metabolic process | 1 |
| GO:0033542 | fatty acid beta-oxidation, unsaturated, even number | 1 |
| GO:0033591 | response to L-ascorbic acid | 1 |
| GO:0033615 | mitochondrial proton-transporting ATP synthase complex assembly | 1 |
| GO:0034052 | positive regulation of plant-type hypersensitive response | 1 |
| GO:0034221 | fungal-type cell wall chitin biosynthetic process | 1 |
| GO:0034227 | tRNA thio-modification | 1 |
| GO:0034314 | Arp2/3 complex-mediated actin nucleation | 1 |
| GO:0034394 | protein localization at cell surface | 1 |
| GO:0034501 | protein localization to kinetochore | 1 |
| GO:0034629 | cellular protein complex localization | 1 |
| GO:0034720 | histone H3-K4 demethylation | 1 |
| GO:0034971 | histone H3-R17 methylation | 1 |
| GO:0035023 | regulation of Rho protein signal transduction | 1 |
| GO:0035067 | negative regulation of histone acetylation | 1 |
| GO:0035278 | negative regulation of translation involved in gene silencing by miRNA | 1 |
| GO:0035304 | regulation of protein dephosphorylation | 1 |
| GO:0035349 | coenzyme A transmembrane transport | 1 |
| GO:0035456 | response to interferon-beta | 1 |
| GO:0035524 | proline transmembrane transport | 1 |
| GO:0035690 | cellular response to drug | 1 |
| GO:0040029 | regulation of gene expression, epigenetic | 1 |
| GO:0042073 | intraflagellar transport | 1 |
| GO:0042118 | endothelial cell activation | 1 |
| GO:0042128 | nitrate assimilation | 1 |
| GO:0042149 | cellular response to glucose starvation | 1 |
| GO:0042218 | 1-aminocyclopropane-1-carboxylate biosynthetic process | 1 |
| GO:0042255 | ribosome assembly | 1 |
| GO:0042274 | ribosomal small subunit biogenesis | 1 |
| GO:0042350 | GDP-L-fucose biosynthetic process | 1 |
| GO:0042398 | cellular modified amino acid biosynthetic process | 1 |
| GO:0042438 | melanin biosynthetic process | 1 |
| GO:0042450 | arginine biosynthetic process via ornithine | 1 |
| GO:0042493 | response to drug | 1 |
| GO:0042550 | photosystem I stabilization | 1 |
| GO:0042558 | pteridine-containing compound metabolic process | 1 |
| GO:0042617 | paclitaxel biosynthetic process | 1 |
| GO:0042726 | flavin-containing compound metabolic process | 1 |
| GO:0042754 | negative regulation of circadian rhythm | 1 |
| GO:0042779 | tRNA 3'-trailer cleavage | 1 |
| GO:0042780 | tRNA 3'-end processing | 1 |
| GO:0042819 | vitamin B6 biosynthetic process | 1 |
| GO:0043044 | ATP-dependent chromatin remodeling | 1 |
| GO:0043065 | positive regulation of apoptotic process | 1 |
| GO:0043091 | L-arginine import | 1 |
| GO:0043144 | snoRNA processing | 1 |
| GO:0043148 | mitotic spindle stabilization | 1 |
| GO:0043182 | vacuolar sequestering of sodium ion | 1 |
| GO:0043268 | positive regulation of potassium ion transport | 1 |
| GO:0043269 | regulation of ion transport | 1 |
| GO:0043419 | urea catabolic process | 1 |
| GO:0043572 | plastid fission | 1 |
| GO:0043609 | regulation of carbon utilization | 1 |
| GO:0043686 | co-translational protein modification | 1 |
| GO:0043687 | post-translational protein modification | 1 |
| GO:0043952 | protein transport by the Sec complex | 1 |
| GO:0043972 | histone H3-K23 acetylation | 1 |
| GO:0044011 | single-species biofilm formation on inanimate substrate | 1 |
| GO:0044090 | positive regulation of vacuole organization | 1 |
| GO:0044154 | histone H3-K14 acetylation | 1 |
| GO:0044210 | 'de novo' CTP biosynthetic process | 1 |
| GO:0044241 | lipid digestion | 1 |
| GO:0044267 | cellular protein metabolic process | 1 |
| GO:0044272 | sulfur compound biosynthetic process | 1 |
| GO:0045017 | glycerolipid biosynthetic process | 1 |
| GO:0045039 | protein import into mitochondrial inner membrane | 1 |
| GO:0045110 | intermediate filament bundle assembly | 1 |
| GO:0045116 | protein neddylation | 1 |
| GO:0045226 | extracellular polysaccharide biosynthetic process | 1 |
| GO:0045487 | gibberellin catabolic process | 1 |
| GO:0045694 | regulation of embryo sac egg cell differentiation | 1 |
| GO:0045730 | respiratory burst | 1 |
| GO:0045732 | positive regulation of protein catabolic process | 1 |
| GO:0045733 | acetate catabolic process | 1 |
| GO:0045736 | negative regulation of cyclin-dependent protein kinase activity | 1 |
| GO:0045737 | positive regulation of cyclin-dependent protein kinase activity | 1 |
| GO:0045843 | negative regulation of striated muscle tissue development | 1 |
| GO:0045900 | negative regulation of translational elongation | 1 |
| GO:0045926 | negative regulation of growth | 1 |
| GO:0045931 | positive regulation of mitotic cell cycle | 1 |
| GO:0045943 | positive regulation of transcription from RNA polymerase I promoter | 1 |
| GO:0046034 | ATP metabolic process | 1 |
| GO:0046148 | pigment biosynthetic process | 1 |
| GO:0046203 | spermidine catabolic process | 1 |
| GO:0046398 | UDP-glucuronate metabolic process | 1 |
| GO:0046471 | phosphatidylglycerol metabolic process | 1 |
| GO:0046477 | glycosylceramide catabolic process | 1 |
| GO:0046512 | sphingosine biosynthetic process | 1 |
| GO:0046548 | retinal rod cell development | 1 |
| GO:0046578 | regulation of Ras protein signal transduction | 1 |
| GO:0046621 | negative regulation of organ growth | 1 |
| GO:0046685 | response to arsenic-containing substance | 1 |
| GO:0046901 | tetrahydrofolylpolyglutamate biosynthetic process | 1 |
| GO:0046967 | cytosol to ER transport | 1 |
| GO:0048015 | phosphatidylinositol-mediated signaling | 1 |
| GO:0048034 | heme O biosynthetic process | 1 |
| GO:0048227 | plasma membrane to endosome transport | 1 |
| GO:0048438 | floral whorl development | 1 |
| GO:0048443 | stamen development | 1 |
| GO:0048444 | floral organ morphogenesis | 1 |
| GO:0048449 | floral organ formation | 1 |
| GO:0048462 | carpel formation | 1 |
| GO:0048497 | maintenance of floral organ identity | 1 |
| GO:0048608 | reproductive structure development | 1 |
| GO:0048638 | regulation of developmental growth | 1 |
| GO:0048833 | specification of floral organ number | 1 |
| GO:0050667 | homocysteine metabolic process | 1 |
| GO:0050688 | regulation of defense response to virus | 1 |
| GO:0051225 | spindle assembly | 1 |
| GO:0051382 | kinetochore assembly | 1 |
| GO:0051455 | attachment of spindle microtubules to kinetochore involved in homologous chromosome segregation | 1 |
| GO:0051501 | diterpene phytoalexin metabolic process | 1 |
| GO:0051504 | diterpene phytoalexin precursor biosynthetic process pathway | 1 |
| GO:0051513 | regulation of monopolar cell growth | 1 |
| GO:0051553 | flavone biosynthetic process | 1 |
| GO:0051592 | response to calcium ion | 1 |
| GO:0051596 | methylglyoxal catabolic process | 1 |
| GO:0051781 | positive regulation of cell division | 1 |
| GO:0051782 | negative regulation of cell division | 1 |
| GO:0052541 | plant-type cell wall cellulose metabolic process | 1 |
| GO:0052544 | defense response by callose deposition in cell wall | 1 |
| GO:0052837 | thiazole biosynthetic process | 1 |
| GO:0055070 | copper ion homeostasis | 1 |
| GO:0055072 | iron ion homeostasis | 1 |
| GO:0055078 | sodium ion homeostasis | 1 |
| GO:0055091 | phospholipid homeostasis | 1 |
| GO:0055122 | response to very low light intensity stimulus | 1 |
| GO:0060003 | copper ion export | 1 |
| GO:0060154 | cellular process regulating host cell cycle in response to virus | 1 |
| GO:0060321 | acceptance of pollen | 1 |
| GO:0060862 | negative regulation of floral organ abscission | 1 |
| GO:0060919 | auxin influx | 1 |
| GO:0061077 | chaperone-mediated protein folding | 1 |
| GO:0070234 | positive regulation of T cell apoptosis | 1 |
| GO:0070370 | cellular heat acclimation | 1 |
| GO:0070526 | threonylcarbamoyladenosine biosynthetic process | 1 |
| GO:0070536 | protein K63-linked deubiquitination | 1 |
| GO:0070887 | cellular response to chemical stimulus | 1 |
| GO:0070918 | production of small RNA involved in gene silencing by RNA | 1 |
| GO:0070919 | production of siRNA involved in chromatin silencing by small RNA | 1 |
| GO:0071044 | histone mRNA catabolic process | 1 |
| GO:0071076 | RNA 3' uridylation | 1 |
| GO:0071216 | cellular response to biotic stimulus | 1 |
| GO:0071277 | cellular response to calcium ion | 1 |
| GO:0071395 | cellular response to jasmonic acid stimulus | 1 |
| GO:0071422 | succinate transmembrane transport | 1 |
| GO:0071484 | cellular response to light intensity | 1 |
| GO:0071493 | cellular response to UV-B | 1 |
| GO:0071668 | plant-type cell wall assembly | 1 |
| GO:0071733 | transcriptional activation by promoter-enhancer looping | 1 |
| GO:0071806 | protein transmembrane transport | 1 |
| GO:0071918 | urea transmembrane transport | 1 |
| GO:0072332 | signal transduction by p53 class mediator resulting in induction of apoptosis | 1 |
| GO:0072489 | methylammonium transmembrane transport | 1 |
| GO:0072511 | divalent inorganic cation transport | 1 |
| GO:0072661 | protein targeting to plasma membrane | 1 |
| GO:0080005 | photosystem stoichiometry adjustment | 1 |
| GO:0080024 | indolebutyric acid metabolic process | 1 |
| GO:0080037 | negative regulation of cytokinin mediated signaling pathway | 1 |
| GO:0080110 | sporopollenin biosynthetic process | 1 |
| GO:0080119 | ER body organization | 1 |
| GO:0080140 | regulation of jasmonic acid metabolic process | 1 |
| GO:0080143 | regulation of amino acid export | 1 |
| GO:0080158 | chloroplast ribulose bisphosphate carboxylase complex biogenesis | 1 |
| GO:0080163 | regulation of protein serine/threonine phosphatase activity | 1 |
| GO:0080172 | petal epidermis patterning | 1 |
| GO:0080175 | phragmoplast microtubule organization | 1 |
| GO:0080182 | histone H3-K4 trimethylation | 1 |
| GO:0090071 | negative regulation of ribosome biogenesis | 1 |
| GO:0090316 | positive regulation of intracellular protein transport | 1 |
| GO:0090322 | regulation of superoxide metabolic process | 1 |
| GO:0097193 | intrinsic apoptotic signaling pathway | 1 |
| GO:2000023 | regulation of lateral root development | 1 |
| GO:2000033 | regulation of seed dormancy | 1 |
| GO:2000122 | negative regulation of stomatal complex development | 1 |
| GO:2000280 | regulation of root development | 1 |
| GO:2000652 | regulation of secondary cell wall biogenesis | 1 |
| GO:2000694 | regulation of phragmoplast microtubule organization | 1 |
| GO:2000904 | regulation of starch metabolic process | 1 |
| GO:2001142 | nicotinate transport | 1 |
| GO:2001143 | N-methylnicotinate transport | 1 |
